# Analysis and mitigation of PQ disturbances in grid connected system using fuzzy logic based IUPQC

**DOI:** 10.1038/s41598-023-49042-z

**Published:** 2023-12-16

**Authors:** TatiReddy Ravi, K. Sathish Kumar, C. Dhanamjayulu, Baseem Khan, K Rajalakshmi

**Affiliations:** 1 School of Electrical Engineering, Vellore Institute of Technology, Vellore, Tamil Nadu 632014 India; 2 Department of Electrical and Computer Engineering, Hawassa University, 05 Hawassa, Ethiopia; 3 Department of EEE, Kings Engineering College, Chennai, Tamil Nadu 602117 India

**Keywords:** Energy science and technology, Engineering

## Abstract

Renewable energy integration introduces grid instability due to variable and intermittent sources like solar and wind, impacting reliability. This paper provides a thorough discussion of recent advancements and emerging trends in grid-integrated wind energy systems (GIWES) and grid-integrated solar energy systems (GISES). More than 70 research articles have been rigorously assessed and listed the technological and economic challenges. The increase in installations of grid-Integrating systems gives rise to challenges like as grid strain, peak shaving impacts, unpredictability of renewable energy sources (RES), and power quality disturbances. A variety of custom power devices, such as dynamic voltage restorers (DVR), static synchronous compensators (STATCOM), active power filters (APF), and unified power quality conditioners (UPQC), have gained popularity in response to these challenges. Among the various challenges, power quality disturbances, including voltage sag, swell, current and harmonics pose significant issues. To address these disturbances this work present a novel approach utilizing fuzzy logic (FL) to develop multi-feeder interline unified power-quality conditioners (MF-IUPQCs). The MF-IUPQC has three legs and three levels, each of which has four diode-clamped inverters. Switching is carried out through the use of space vector pulse width/duration modulation (SVPWM). Total harmonic distortion (THD) induced by nonlinear loads is reduced by the FLC-based MF-IUPQC, which also improves dynamic performance and offers a smooth DC-link voltage. The proposed control mechanism is implemented using MATLAB/Simulink. The fuzzy-based controller is compared to the industry-standard proportional-integral (PI) controller to determine its efficacy. Among them, the MF-IUPQC based on FLC delivers the smoothest voltage profile and the lowest THD.

## Introduction

### Background and motivation

**Figure 1:**
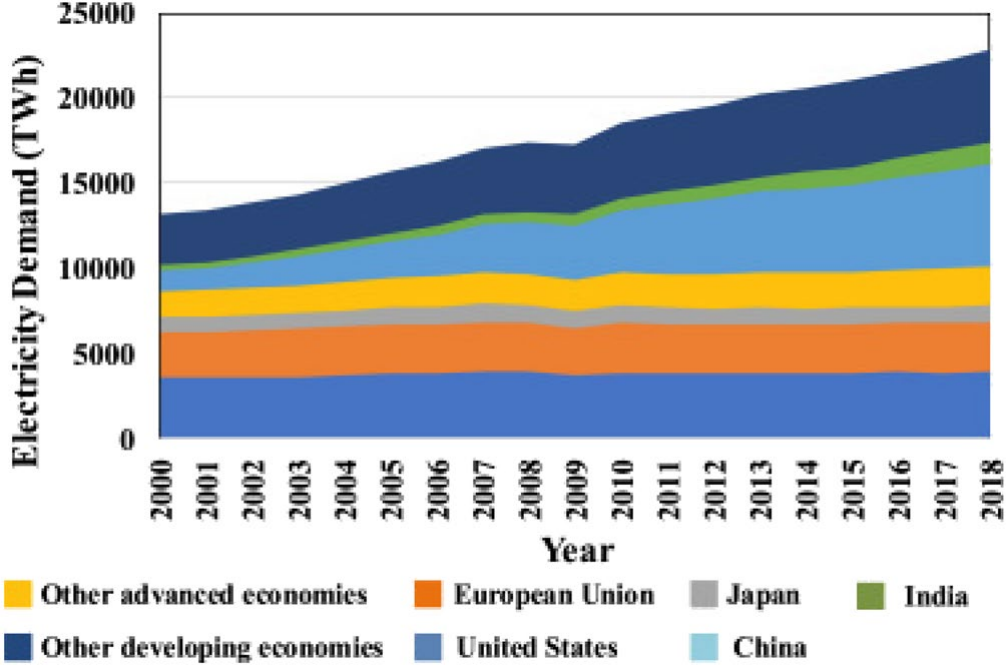
Global electricity demand by region^1^.

**Figure 2:**
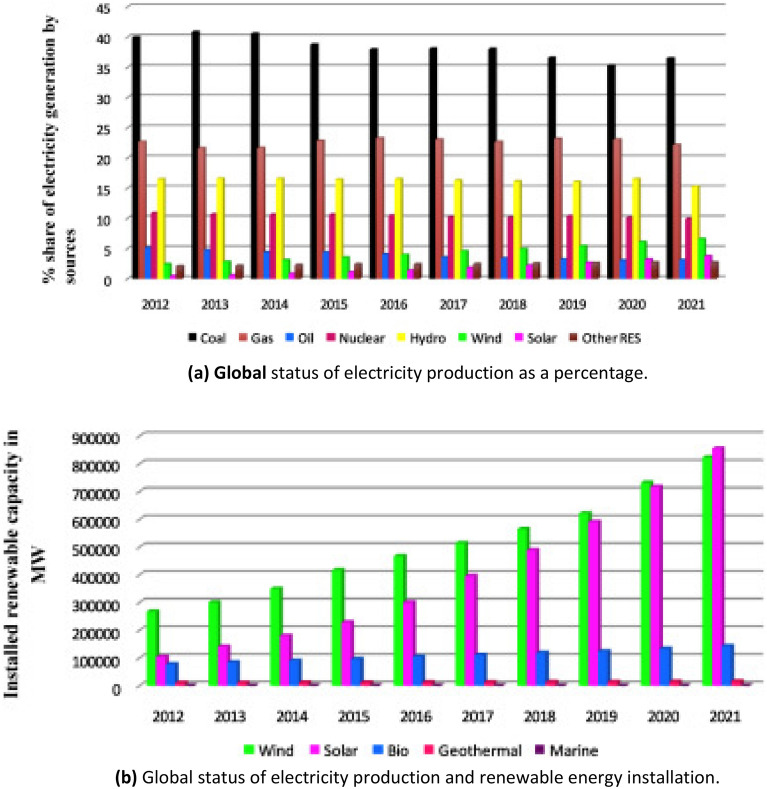
(**a**) Global status of electricity production as a percentage. (**b**) Global status of electricity production and renewable energy installation.

Over the past three decades, the worldwide demand for energy has increased dramatically and steadily, as illustrated in Fig. 1 (a 72% rise between 2000 and 2018). World electricity consumption increased beyond 23,000 TWh by the end of 2018, a phenomenal rise of more than 4% from the previous year's level. Countries with rapidly developing economies, rising populations, and higher per capita incomes are mostly responsible for this increase in demand. As a result, the electrical needs of these countries have increased dramatically, contributing significantly to the worldwide shortage^1^. In 2018, yearly worldwide power usage was roughly 157,064 TWh, with 86% of this total coming from fossil fuels. Unless considerable efforts are undertaken to move towards decarbonization, continuing to rely heavily on fossil fuels will result in the emission of about 35 Gt/y of CO_2_, creating significant environmental dangers. The percentage of electricity generated from various sources from 2012 to 2021 is shown in Fig. 2a A more sustainable energy sector in the future can be achieved through the increased usage of renewable energy in power generation. Wind and solar power generation facilities are particularly promising because of their limitless availability, large power supply capacities, and cost competitiveness, among other advantages^2^. Figure 2b illustrates the global landscape of renewable energy installations measured in megawatts (MW). Wind as well as solar energy sources are irregular at various timescales varying from minutes to years owing to reliance on climate condition^3^, which inflict confronts to the nationwide electrical grid workers. Differences of these two resources doesn't have a similar feature, also frequently, the dissimilarity of wind as well as solar sources are not adjusting in relating to a phase frequency construction in addition to magnitude of variation^4^. Hence, an appropriate merge of these two sources can be an approach to achieve, such as, a partly smoothed overall output power^5^. Variability along with intermittent of these two sources may be well controlled as well as visualized once these schemes are utilized simultaneously^6^. In power system having a considerable contribution of these two sources, it is essential to know relationship among power resources to go with customers necessities and improve spinning reserve^7^.

The integration of photovoltaic (PV) and wind energy generation into the grid presents several challenges, including the generation of intermittent energy, problems with grid integration, a load on grid capacity, power quality disruptions, management complications, and the requirement for supportive regulatory frameworks and market mechanisms. These challenges are brought about by the intermittent nature of renewable energy sources (RES), the requirement for grid reinforcement, concerns over power quality, the balancing of supply and demand, and the guaranteeing of appropriate pay for producers of renewable energy. In order to effectively address these difficulties, rigorous planning, advanced forecasts, grid control systems, and supportive legislation are required.

### Contribution and organization

This paper presents several significant contributions: It provides a thorough examination of the trends and challenges encountered in grid-integrated wind and solar energy systems over the past decade. To address some major PQ disturbances like voltage fluctuations, current and voltage harmonics this paper introduces a new Multi-Feeder Interline Unified Power Quality Conditioner (MF-IUPQC) based on a Fuzzy Logic Controller (FLC) with a significant impact on power quality enhancement. It improves the waveforms of voltage and current, reduces harmonics, regulates voltage fluctuations, and manages load currents. This improves power supply stability and dependability, resulting in less downtime, equipment wear, and operational costs. The MF-IUPQC quick response to voltage disturbances, current balancing, harmonic suppression, and DC-link voltage stability all work together to improve system dynamic performance. This results in improved power quality and dependability, lowering the risk of equipment damage and operational disruptions. The total harmonic distortion (THD) is effectively reduced below the permissible limit of 5% as specified in the IEEE 519-1992 standard. The paper is structured as follows:

1. Section "Literature review" presents a literature review of the trends and challenges associated with grid-connected solar and wind energy systems.
2. Section "Proposed power quality mitigation technique" details the proposed technique for improving power quality (PQ).
3. Section "Results and discussion" provides an in-depth analysis of the simulation results and initiates a discussion.
4. Lastly, section "Conclusion" concludes the paper, summarizing the key findings and contributions.

Overall, this article significantly contributes to the understanding of grid-integrated renewable energy systems. The introduction of the FLC-based MF-IUPQC system offers a promising solution for effectively addressing power quality issues. The simulation results validate the system's performance, showcasing its ability to meet the required standards and improve PQ in the grid.

## Literature review

In recent years, emissions from nonrenewable energy sources have significantly contributed to global warming. Consequently, industries and governments are actively researching alternative energy sources to mitigate these negative environmental effects. Nonconventional energy sources, often known as alternative energy sources, refer to forms of energy that are distinct from traditional fossil fuels such as coal, oil, and natural gas. Wind and solar energy^8^ have emerged as indispensable elements within the energy generation domain, particularly in view of the depletion of fossil fuel supplies. Wind and solar energy offer significant advantages, such as environmental tolerance and abundant availability, among alternative energy sources. Nonconventional energy-source power generation technologies play a crucial role in advancing renewable energy knowledge and reducing dependence on fossil fuels. Biomass, geothermal, ocean, micro hydro, tidal, wind, and solar energy are among the available power generation technologies. However, wind and solar resources are favored due to their abundance and adaptability to a variety of conditions and locations, even in remote areas where constructing transmission lines is difficult^8,9^. Even though these resources are improving in numerous ways, they have certain limitations, such as a behavior that continuously varies in response to changes in ecological conditions. Despite their benefits, wind and solar energy have drawbacks, predominantly due to their inherent variability in response to changing environmental conditions. This variation can result in power fluctuations and an inability to satisfy load demand, reducing the overall efficiency of the system. Moreover, depending only on these resources may result in excessive and costly system designs. It is crucial to integrate wind and solar energy sources with adequate system designs in order to overcome the difficulties caused by the fluctuating behaviour of these energy sources. By implementing a suitable system arrangement, it is possible to address the complexity caused on by these energy sources' continually varying behavior^10^, and this configuration can compensate the disadvantage of a single source by making use of additional source strength. As a result, a hybrid system that combines solar and wind power with a battery storage system can be seen as a feasible choice, especially in nations like India where environmental issues are crucial to economic development. Numerous hybrid energy systems present a feasible option for mitigating the persistent unpredictability associated with different renewable energy sources, hence producing a more consistent and reliable electricity supply. The utilisation of energy storage enables the storage of surplus energy generated during periods of plenty of resources, facilitating its subsequent utilisation during periods of lack of resources. This enhancement contributes to the overall stability of the system and facilitates the consistent fulfilment of demand^11^. Hence, the use of wind and solar energy-based hybrid systems integrated with battery storage is a feasible solution to address the inherent challenges associated with the intermittent nature of these renewable energy sources. By integrating multiple sources and incorporating energy storage, these systems can provide a reliable and continuous power supply while considering environmental impacts in the aim of sustainable economic development, especially in countries like India.

### Recent advancements and emerging trends in grid integrated wind energy systems (GIWES)

**Figure 3:**
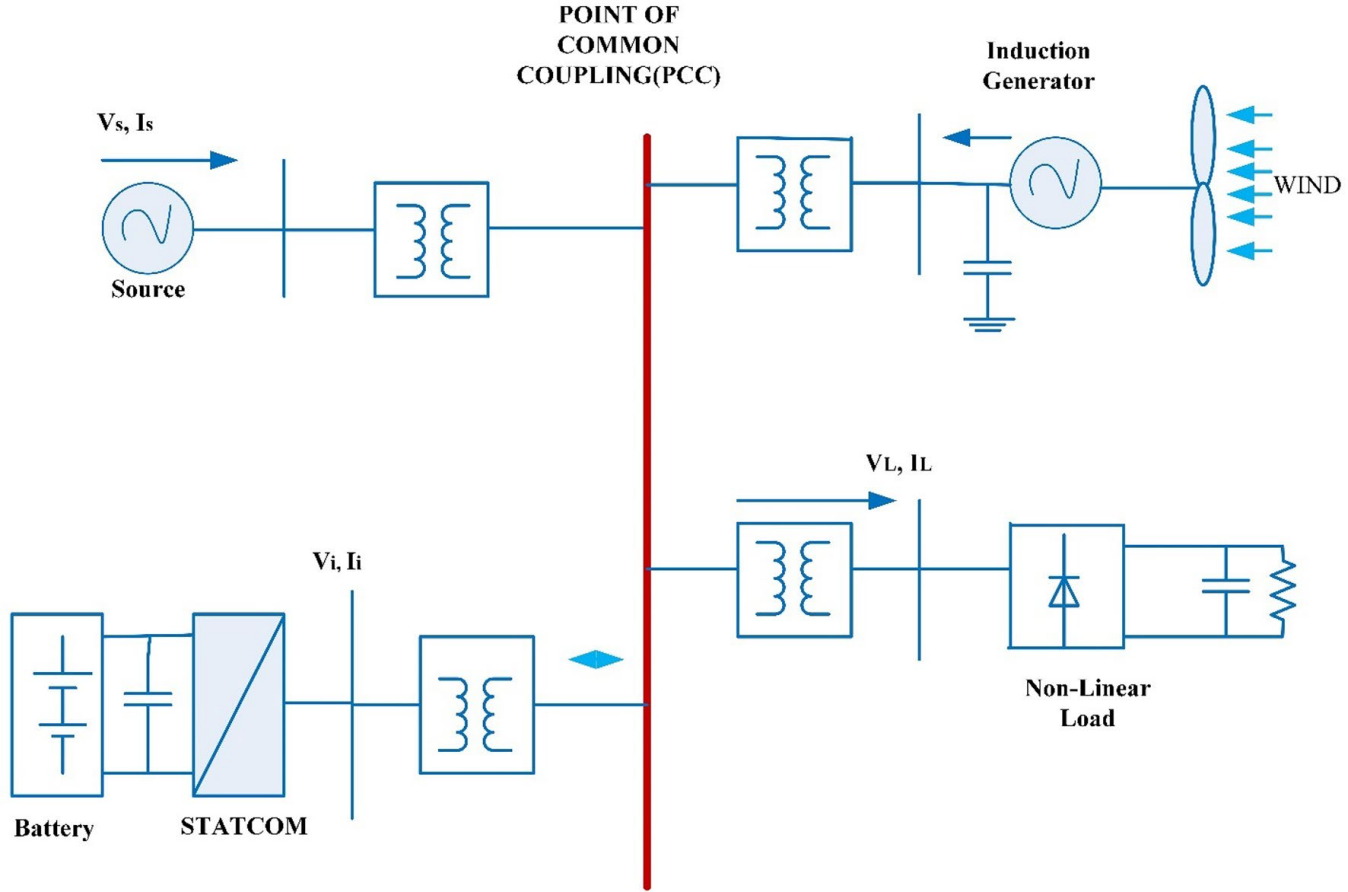
Wind energy system linked to grid.

The wind system that is linked to grid for enhancing the quality of power at point of common coupling (PCC) is as shown in Fig. 3. The scheme which is associated to grid includes a wind system and battery system which stored the energy with static compensator (STATCOM).

Meeting the growing demand for power due to population growth and greater usage was a big concern. The utilisation of renewable energy, particularly wind power, as the predominant energy source for distribution has prompted apprehensions over power system reliability and quality^12^. Power electronics-based forced commutated converters were added to the distribution system to overcome these challenges and assure stable and dependable operation while improving power quality at the PCC. One major source of concern was the current distortion generated by nonlinear loads, which could result in voltage distortions and, in extreme situations, serious consequences for the power system. It was difficult yet critical to identify and address such complex power system challenges, particularly when incorporating wind energy systems into the grid^13^. Control and regulation of factors such as voltage magnitude, transmission impedance, and load angle are required to alleviate these difficulties and improve system efficiency^14^.

Wind power generation has become an essential source of renewable energy, necessitating an increase in transmission capacity and an improvement in system dependability. As wind energy integration increased, it became necessary to address system stability, security, functionality, and control^15^. Maintaining the VAR (Volt-Ampere Reactive) balance between the wind system and electricity systems to keep the voltage at PCC within prescribed limits, especially during voltage dips, to accomplish low voltage ride-through (LVRT), was one of the challenges of this integration. The study conducted by Basit et al. focused on the examination of renewable energy sources (RES) and their potential for enhancing durability, efficiency, and energy capacity through the use of energy storage systems (ESSs). The investigation also included the examination of efficiency, harmonics, and inertia in photovoltaic (PV) systems. Their study is important because it uses multiple FACTS controllers to look into flexible alternating current transmission systems (FACTS) in the context of renewable energy power networks. The researchers created three different models and afterwards made the observation that the utilisation of FACTS devices contributes to the stabilisation of integrated power networks based on RES^16^. In a separate study, Ashok Kumar et al. proposed the integration of a STATCOM with a Battery Energy Storage System (BESS) as a means to enhance the flexibility of wind energy and facilitate its integration into the grid. A novel controller was developed for the wind energy configuration and grid-connected STATCOM-BESS system. The employed Simulink model utilised a hysteresis current controller based on pulse width modulation. The use of this controller enables the STATCOM with BESS to effectively regulate VAR, mitigate harmonic distortions, and maintain the stability of source current and voltage while minimising phase angle fluctuations^17^. The authors conducted a study^18^ to examine the challenges related to power system security, PQ, and stability that emerge as a result of the integration of wind power into the electrical grid. It was noticed that the aforementioned consequences exhibit more prominence with the expansion of wind power penetration. The study not only identified the challenges but also proposed several reforms to policy that may effectively address these challenges, leading to enhanced power system flexibility and improved integration of wind power into the grid. The present study included an analysis of the challenges associated with integrating wind energy into the existing system and proposed potential strategies to mitigate these obstacles. The investigation examined various aspects including uncertainty in power generation, power quality, angular and voltage stability, as well as socio-economic and environmental concerns within the energy market. To address the aforementioned challenges, the research^19^ proposes the use of energy storage technologies, wind energy legislation, and grid codes as potential solutions. Aditya et al. conducted a study of various PQDs that are commonly observed in grid-connected systems. The study identified several technological challenges, namely issues pertaining to protection, storage, islanding, reliability, and stability. The article concluded by discussing potential solutions to the PQ problem and addressing the technological challenges associated with grid-connected systems^20^. Youssef et al. developed a novel MPPT method that kept the controller in the optimal region of the power-speed curve. Based on the idea of a multi-sector process, a variable-step perturb and observe (VSPO) MPPT strategy was presented. This technique uses a power versus speed curve and a synthesized curve to split the operational region into four subregions. The correct increment size was associated with the business sector. For two MPP-near regions, the step size was extremely small. The controller on our machine worked in big increments. Machines and grid-side converters make up the large-scale grid-tied wind energy conversion system (WECS). Using MATLAB and Simulink, the suggested system was evaluated on a 1.5 MW grid-connected PMSG-WECS. The strategy outperformed P&O techniques^21^ because of its efficiency and the rapid response time of the system.

In order to enhance the power quality of grid-connected wind projects, the authors of^22^ proposed using a distribution static compensator (DSTATCOM) in conjunction with a BESS. This scheme was developed with the load and induction generator VAR requirements in mind. Simulink was used for both the design and testing of this strategy. In their study, Jayanthi et al. presented the idea of conducting a comparative analysis on the performance of GIWES processed by a doubly fed induction generator (DFIG) with and without the implementation of a crowbar. To simulate various scenarios, the researchers employed a 1.5 MW wind energy conversion system. According to the requirements set by the Indian Grid, it is imperative for WES to ensure the maintenance of synchronization, even in the face of fast fluctuations in voltage and current. At that particular moment, a crowbar resistance was linked to a testing setup, subsequently followed by an Insulated Gate Bipolar Transistor (IGBT). The outcomes obtained from conducting a simulation on a grid-connected DFIG-driven WECS demonstrated the transient behaviour of the grid following the introduction of both Line-to-Ground (LG) and Line-to-Line-to-Ground (LLG) faults. When a grid fault occurred, it concurrently discovered the specific crowbar that mitigated the excessive fault current. Simulink was utilised to facilitate the visual representation of waveforms pertaining to several problem scenarios^23^. The hybrid concept, as described by Panigrahi et al., establishes a connection between PV and wind energy systems with the electrical grid. Additionally, the article included a description of PV cells and highlighted their distinctive characteristics. The discourse also encompassed the stages incorporated in the process of integrating wind power into the electrical grid, as well as the various characteristics that necessitate synchronisation in order to achieve successful integration. The measurements and analysis include wind velocity, voltage, reactive power, and active power. This study has contributed to the development of wind and PV source models and their integration with the electrical grid. The validity of the findings was confirmed through the utilisation of MATLAB^24^. The hybrid renewable energy systems (HRES) examined by Srinivas et al. contain a combination of wind, solar PV, and battery storage. The assessment covers topics such as energy management, battery charging and discharging management, as well as various power electronics topologies and control methods. The paper underscored the necessity for further investigation of HRES^25^ and thoroughly analyzed the opportunities for future developments. Phei et al. provided an overview of the application of matrix converters in grid-connected wind turbine systems based on PMSGs. The system was employed in small-scale wind turbine (WT) applications, namely in the context of conservational housing. It contained a wind turbine, PMSG, and a three-phase matrix converter. The verification of the system's performance and cooperation was conducted through the analysis of simulation data^26^. The PQDs caused by GIWES were examined by Ndirangu et al. This research focused on the examination of reduction methods that fall within the limits of human control. In order to address the challenges caused by the implementation of WECS, scholars have proposed possible directions for future research^27^. Amita et al. utilised wind power, which is considered an alternative kind of energy, to produce electrical energy. The PQ of GIWES with nonlinear loads was enhanced with the implementation of a unified power quality conditioner (UPQC). A concise description of the issues pertaining to PQ was provided in order to facilitate potential enhancements. This study successfully demonstrated the enhancement of PQ in GIWES through the use of both STATCOM and a UPQC. The STATCOM device is responsible for regulating the reactive power (VARs) and maintaining the synchronisation of the source voltage and current. The regulation of power quality, which includes both actual power and volt-ampere reactive (VAR) power, was achieved through the utilisation of UPQC. The controller made adjustments to the voltage or current flowing through the system in response to variations in wind speed. The aforementioned modification resulted in an enhancement of the system's power factor without considering losses, hence increasing its overall efficiency. In order to enhance the PF of the system, MATLAB/Simulink models were developed. These models incorporated a STATCOM along with a wind system connected to a UPQC. The findings from the simulations indicated that the system exhibited considerably improved performance while utilising UPQC compared to STATCOM^28^. In^29^ Daryabi et al. proposed the integration of a STATCOM controller with a TSC to enhance power quality, hence introducing the idea of GCWES. This article also presented challenges related to power quality, examined the repercussions of these issues on customers, and put forward possible solutions from the perspective of electric utility providers. Hence, the mitigation of current distortion is achieved, resulting in improved phase synchronisation between voltage and current. This necessitates the implementation of PF correction and an adjustment of the voltage-current ratio for both the wind generator and the load at the PCC. Hence, the utilisation of wind generation in combination with the FACTS device has shown significant efficacy in maintaining the desired PQ profile. The functions for GIWES utilising STATCOM were created and implemented using Matlab and Simulink. According to Magesh et al., the performance of grid-connected PMSG-VSWT can be enhanced through the utilisation of PI controllers based on the Golden Eagle Optimisation Algorithm (GEOA). The real-time wind farm data obtained from Tamil Nadu, India, demonstrate superior performance in comparison to the controllers Newton Raphson (NR)-PI, PSO, and Grasshopper Optimisation Algorithm (GOA). This approach optimises grid power generation by effectively managing unexpected fluctuations. The adaptability of GEOA controllers provides many benefits to microgrids, smart grids, EV charging stations, and green energy storage^30^.In a separate study, Rawa et al. utilised a series compensator known as a Dynamic Voltage Restorer (DVR) to address PQDs caused by RESs, such as PV and wind farms. The utilisation of the Gorilla Troops Algorithm (GTA) in conjunction with proportional-integral (PI) controllers in DVRs has been found to effectively address voltage quality issues like sag, swell, flashing, and harmonics^31^.

Feyzi et al. presented the sliding mode control strategy they developed specifically for WECS based on PMSG in reference^32^. This control methodology is suitable for both typical operational scenarios and instances characterized by grid faults. The researchers utilised a methodology that involved modifying the controllers on both the machine-side and grid-side. During instances of grid faults, the machine-side controller modifies the dc-link voltage, diverting its attention from the regulation of reactive and active power. Ali et al. presented a finite-control set model predictive control methodology for WECS employing a PMSG in their paper^33^. The primary goal of this technology is to enhance the LVRT capability and maintain the stability of the DC link voltage, particularly when dealing with both asymmetrical and symmetrical grid issues. The objective of the proposed methodology is to improve the effectiveness of state switching and provide support for grid voltage by injecting reactive power during occurrences of voltage sags. The model-predictive control strategy for the management of a crowbar circuit in a PMSG-based wind system was proposed by the authors of reference^34^. The crowbar circuit design incorporates the use of supercapacitors and resistors to successfully reduce changes in DC voltage. The control methodology contains the capacity to dynamically adjust the resistance value with the aim of stabilizing the DC voltage, resulting in a smoother functioning of the system. In their study, Islam et al.^35^ introduced a novel approach to enhance the LVRT capabilities of doubly fed induction generator-based wind energy conversion systems (DFIG-WECS). This approach involves the use of fault current limiters equipped with saturated amorphous alloy cores. The objective of this work was to enhance LVRT capabilities. The newly developed fault current limiter exhibits a reduced impedance under normal operating conditions and requires a lower DC excitation current compared to traditional cores. The core undergoes desaturation as the voltage decreases to extremely low levels, resulting in an increase in impedance, a reduction in fault currents, and ultimately an enhancement of the LVRT capability.

In^36^, Song, Yuyan, et al. proposed a Q-learning-based robust model predictive control strategy for DFIGs as a means of mitigating rotor overcurrent and preventing frequent activation of the crowbar circuit in the event of grid disturbances. R. Hiremath and T. Moger introduced a modified super-twisting technique that utilizes second order sliding mode control in Ref.^37^. The utilisation of sophisticated phase-locked loop methodologies, although highly efficient, frequently necessitates significant computational capabilities, resulting in processing delays. In order to address this issue, there has been an increasing recognition of the effectiveness of second-order generalized integrators (SOGI) and adaptive notch filters (ANF) in the extraction of fundamental signals and the reduction of harmonics and disturbances. Moreover, frequency-locked loops (FLLs) have demonstrated significant efficacy in demanding grid scenarios. The FLLs utilize a nonlinear feedback architecture in order to extract the fundamental signal by means of a filter and effectively adjust to fluctuations in frequency^38^. The variation known as SOGI-FLL combines the concepts of SOGI and integrated controller capabilities to provide frequency regulation. Nevertheless, the application of linear control in frequency estimation is subject to some constraints. In order to tackle the issues of harmonic rejection, DC bias reduction, and frequency variation, innovative control methods such as enhanced SOGI, improved ANF, and higher-order techniques have been presented in^39^.In the study conducted by the authors, a multi-mode control technique is introduced for systems that combine wind and battery technologies^40^. This strategy provides a streamlined and less intricate architectural design, leading to enhanced efficiency in execution speed. Nevertheless, its primary use is limited to DC microgrids. Using a fractional order FLC, Krishna et al.^41^ presented a UPQC for handling power quality issues. In a power distribution system with non-linear loads, they proved UPQC efficacy. There were four types of controllers used in their research: a fuzzy logic controller, an adaptive FLC, a fractional order FLC, and a fuzzy output phase inverter. However, this method was not without its flaws, such as its cumbersome dimensions, resonant problems, and lengthy procedures. A UPQC system was further developed by Krishna et al.^42^ to deal with a wider variety of power quality challenges. To minimize voltage distortion, a series active power filter (APF) and a flexible FLC were used in this setup. The DC interface voltage was maintained by the variable FLC, while the shunt APF was controlled by the load current through the DQ axis. While this model did enhance performance, it was computationally intensive and had limited precision in terms of compensator and controller parameters, leading to less than ideal results in some cases. In^43^ Power system concerns like swell, sag, interruptions, and harmonics were addressed with an intelligent controller-based compensation model. Low power factor, load imbalances, voltage changes, and harmonics cause these concerns. A DFIG with a grid-connected nonlinear load system tests the proposed model's deep learning-based intelligent controller to tackle these obstacles. The controller injects electricity during issues to stabilize power flows. UPQC reduced harmonics and improved power quality the best among four load voltage compensators: DPFC, USSC, UPFC, and UPQC. Advanced compensators using new technology may be studied for comprehensive issue mitigation. In a broader sense the investigation highlights the increasing importance of wind power generation as a sustainable form of energy, resulting in the need for the expansion of transmission capacity and improvements in system dependability. The successful integration of wind energy requires the development of strategies that effectively address issues related to stability, security, functionality, and control. The preservation of Volt-Ampere Reactive (VAR) equilibrium is of greatest significance for voltage regulation, particularly in the context of voltage fluctuations, in order to ensure a smooth transition through low voltage conditions, commonly referred to as low voltage ride-through (LVRT). Numerous research studies explore the potential of energy storage systems (ESS) in enhancing the durability, efficiency, and management of harmonics in renewable energy sources. The effectiveness of implementing devices such as Static Synchronous Compensators (STATCOM), Battery Energy Storage Systems (BESS), Dynamic Voltage Restorer (DVR), and Unified Power Quality Conditioners (UPQC) is evidenced by their contribution to the improvement of grid stability and enhancement of power quality. Subsequent investigations could concentrate the examination of energy storage, regulations, grid codes, and improved control techniques as possible solutions for addressing the challenges related to the integration of wind generation.

### Recent advancements and emerging trends in grid integrated solar energy systems (GISES)

**Figure 4:**
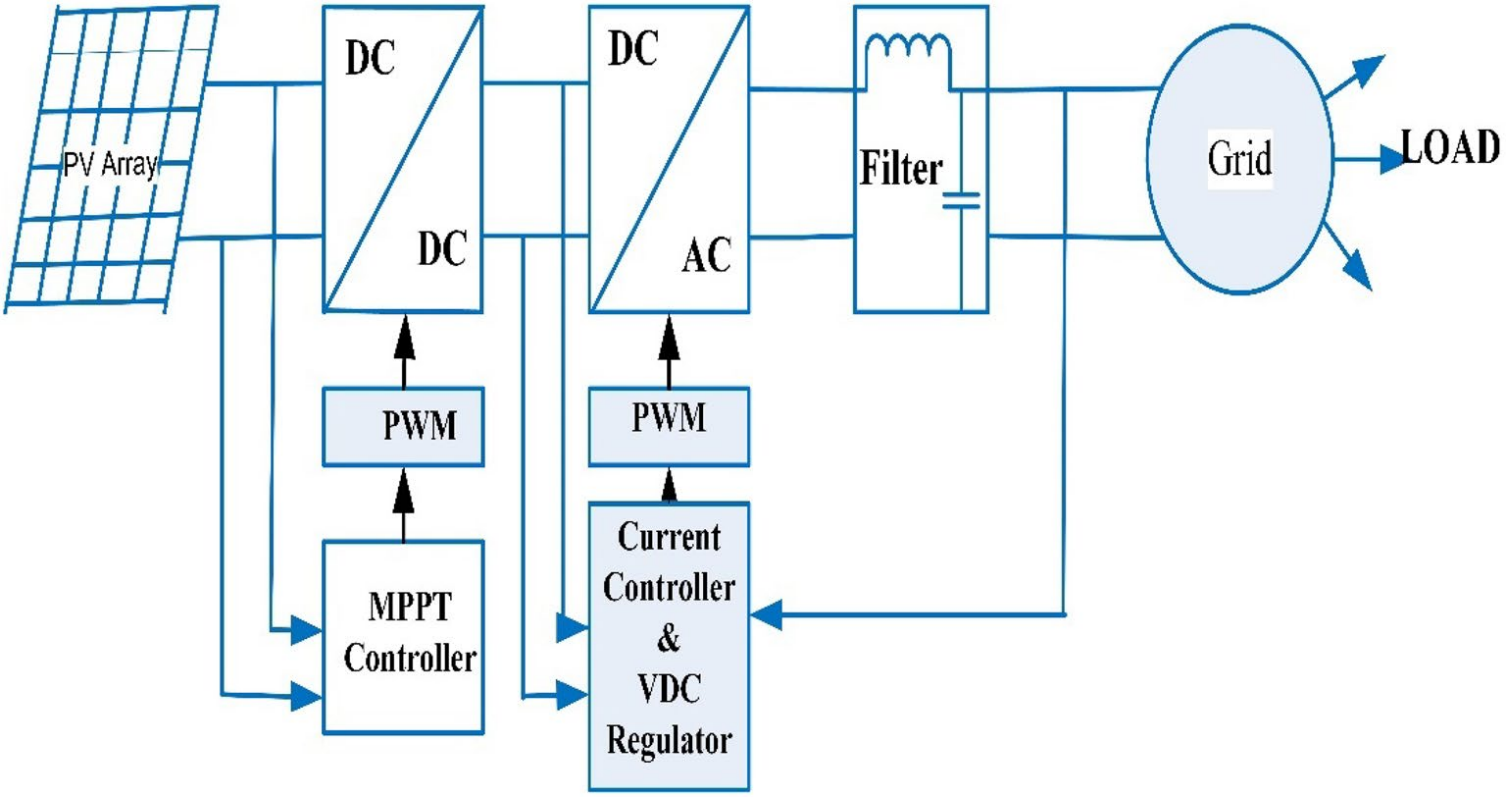
PV array with MPPT controller (linked to grid).

A photovoltaic scheme which linked to grid with maximum power point tracking (MPPT) control is revealed in Fig. 4 The core components of PV system are: PV array (different configurations of modules), junction box, power conditioning unit (dc–dc converter and inverter circuits), pulse width modulation (PWM) circuit, MPPT controller, current controller in addition to DC voltage regulator circuit, and filter. Installations of grid-integrated solar energy systems are growing all over the world. This is because of the demand for renewable energy sources (RES), the falling cost of technology, the ease of access to solar energy, and the improvement of technology for large-scale uses. As the demand for these systems rises, many problems arise when trying to connect them. PV systems usually only produce electricity for a small amount of time during the day, even though the load demand profiles are almost the opposite of the solar energy available. Most GISES have PV panels that convert the energy from the sun into DC power and a transformer with an MPPT that changes the power from DC to AC so that it can be sent to an electricity grid. When energy is exported, the inverter has to meet the standards and needs of the electricity grid. The number of PV installations has grown exponentially, mostly because the cost of the technology has gone down and because governments and utility companies maintain programs that concentrate on GIPVS.

Despite the rapid increase in the number of installations of grid-integrated photovoltaic systems (GIPVS) and their expanding importance in meeting large-scale load requirements, numerous technological and economic issues arise from the increasing number of these systems. The factors to consider include the strain on the utility grid during the shift from high peak generation hours to off-peak generation hours, the phenomenon of peak shaving resulting from generation peaks exceeding the peak load, and the inherent unpredictability associated with installations of GIPVS. In a recent study conducted by Obi et al., an investigation was undertaken to explore the latest advancements in GIPVS. Research was done with the aim of defining standards to effectively handle the current and anticipated technological issues caused by the widespread use of GIPVS. The research examined a range of methodologies, such as transformer-less inverters, solar tracking, and MPPT, with the aim of enhancing the efficiency of PV systems. Additionally, the authors highlighted the significance of inverters in offering ancillary services, like VAR power management, frequency control, and energy storage, as a means to address challenges arising from the extensive use of GIPVS^44^. Colmenar-Santos focused on making grid-connected PV power plants more competitive to meet future demand and resolve grid management challenges. The study underscored the significance of accurate system regulation, particularly in the context of integrating diverse energy sources into an electrical grid. They investigated the requirements of system operators for both fundamental operations and intelligent regulation, as well as the technical knowledge of system components required by network operators^45^.

Ismail et al. conducted a study wherein they presented the implementation of a 60 MW grid-connected 3-phase hybrid system. This system used a Ni–Cd battery for the purpose of storing DC power generated by the PV module. Their inventive design incorporated a two-stage energy conditioning and conversion element. Two Adaptive Neuro-Fuzzy Inference System (ANFIS) controllers were introduced for effective battery management. One controller was responsible for battery charge, while the other was responsible for battery discharging, independent of the grid^46^. Chakir et al. proposed a novel PV battery system that efficiently regulates energy flow by implementing an optimal management procedure when linked to the grid. With a DC bus source connection topology, their hybrid architecture resolved synchronisation problems between sources. Combining charge-limiting power and discharge strategies substantially lengthened the battery's life. Before actual implementation^47^, the dynamic behaviour of system components was simulated using mathematical models, and an energy management strategy was simulated using MATLAB or Simulink. Singh et al. identified significant problems regarding PQDS related to PV systems and their impact on the performance of the electrical grid. The authors emphasized the difficulties that arise from power instability and variations in frequency profiles caused by external factors. Power electronic (PE) devices have been recognized as viable solutions for these issues. In order to mitigate the variations in output of PV systems, researchers have suggested the use of energy storage devices and MPPT controllers. Additionally, improvements in materials and storage equipment have the potential to enhance the performance of grid-integrated PV systems and improve system stability^48^. The MATLAB Simulink system developed by Fomba et al. was designed to compute the quantity of electricity transmitted to the grid, utilising temperature, and irradiance as input parameters. Their model included a solar irradiance metre to measure the amount of sunlight, converting meteorological data into signal inputs. The relationship between output variables, namely current, voltage, and power, has been determined by considering the variations in solar irradiation and temperature. The model provided a comprehensive comprehension of how these factors affected PV module characteristics and energy generation potential at specific locations^49^. Khandelwal et al. provided an in-depth examination of the PQ challenges that occur due to the integration of PV systems with the electrical grid. These challenges include several difficulties like overheating, reduced efficiency, diminished equipment longevity, interruptions in the operational processes, insulation failures, and potential loss of data. However, it may offer challenges to completely eliminating the underlying causes. There have been suggestions to address PQDs, such as improving power supply quality and implementing methods to minimize their impact^50^. In their study, Bahri et al. introduced a nonlinear observer-based control strategy for the integration of PV systems into 3-phase loads and utilities using 3-phase voltage source inverters, eliminating the need for DC–DC converters. The control technique employed in their study involved the utilisation of a backstepping procedure that employed d-q transformations as well as the implementation of a nonlinear state observer to estimate the inverter current. The simulation yielded outstanding dynamic performance under diverse operating conditions^51^. In their research, Hamrouni et al. introduced a control mechanism aimed at enhancing the operational efficiency of a two-stage GIPVS, with a particular focus on mitigating the impact of symmetrical grid voltage fluctuations. The primary goal of their regulatory approach was to mitigate the occurrence of catastrophic failures and enhance the reliability of the system in the case of grid malfunctions. During grid-connected operation, simulations confirmed the system's ability to "ride through" symmetrical voltage fluctuations while maintaining a stable dc-link voltage and reducing harmonic distortion^52^. The study conducted by Hamdan et al. explored the use of supercapacitors in grid-connected PV systems, with a focus on maintaining system stability during grid faults. They employed Perturb and Observe (P&O) MPPT technology and highlighted the importance of SC as a storage device in PV systems, particularly during grid fault scenarios. An approach was developed to ensure continuous charging and discharging while also maintaining a stable DC-link voltage. The efficacy of the control strategy, comprising the Energy Storage System (ESS) supercapacitors, was evaluated using MATLAB/Simulink simulations^53^. Selmi et al. conducted a comprehensive study focusing on the analysis of grid-connected solar installations in the Sultanate of Oman. The objective of this study is to do an analysis of a 5-kWp solar system. It reflects the effect that varying the tilt angle has on the total power extracted in the specified area. The study also provides a method for properly matching PV modules with an appropriate inverter to minimize losses. Additionally, this resource offers a techno-financial evaluation of GIPVS installations, providing it to both individuals and professionals to use in their own investigations. The information presented in this study was gathered via the PVsyst 6.6.3 software application^54^. Gabr et al. proposed conducting an assessment of the impact of GIPVS on Total Harmonic Distortion (THD) inside a practical, low-distribution network. The researchers examined two approaches to mitigate PQDs, including the implementation of filters and the utilisation of switching techniques at significantly reduced current levels. The proposed methods were evaluated and compared for their usability and efficiency using MATLAB/Simulink^55^. Pal et al. proposed the use of a DVR as a potential solution to enhance power quality in GIPVS that serve both grid and three-phase applications. The implementation of the PV generation system involved the use of an incremental conductance MPPT technique. Additionally, a basic DVR configuration was enhanced by incorporating a PV system as an additional DC source^56^. In their comprehensive study, Mahela et al. presented a detailed overview of GIPVS. The objective of their paper^57^ was to provide academicians, designers, and engineers with a comprehensive overview of PV energy and grid integration, including control techniques for both 1-phase and 3-phase inverters. Ray et al. conducted a comprehensive comparison of different cascaded H-Bridge inverter topologies to enhance the performance of grid-connected PV systems. The researchers conducted an examination of both single-phase and three-phase topologies, evaluating several control methodologies, including power balance control, energy balance control, and active VAR power regulation. The study proposed prospective avenues, identified obstacles, and outlined future extensions to improve the performance of large-scale PV systems^58^. In their study, Wang et al. introduced an innovative control system for GIPVS. This control system incorporated a dc/dc converter controller to effectively monitor the dc bus voltage. The PV voltage regulator was specifically developed to establish a connection between the MPPT procedure and the power factor correction (PFC) circuit. The implementation of independent controllers for the dc/dc and dc/ac converters facilitated accurate voltage regulation of the dc/dc converter through the utilisation of a current-mode controller that relies on an uncertainty and disturbance estimator (UDE). In addition, the study suggested that an encapsulated voltage PFC technique could improve the efficacy of the current UDE-based PFC for AC voltage protection. The efficacy of the GIPVS system with fault ride-through capabilities was validated by laboratory experiments^59^. Rao et al. have addressed PQDs, including VAR compensation, balanced load currents, and neutral current compensation, by using a four-leg inverter with hysteresis current control. The inverter's regulation facilitated the supply of supplementary current necessary for sustaining grid demand^60^. Priyadarshi et al. introduced a unified hybrid controller for MPPT utilising firefly asymmetrical fuzzy logic control (FAFLC) methodology. The controller was specifically developed to improve MPPT in varying and changing environmental conditions, considering factors such as sun irradiation, hydrogen consumption, and wind speed. The research findings indicated that the MPPT controller based on the FAFLC method successfully identified the Global Maximum Power Point (GMPP) and achieved superior power tracking performance in dynamic scenarios. The validation of the results was conducted using the dSPACE DS 1104 real-time control board^61^. The GIPVS has been the subject of a diverse array of studies and research endeavors. They include topics like system efficiency enhancement, PQ concerns, control strategies, energy storage, and grid integration. Utilising sophisticated simulation tools such as MATLAB/Simulink and real-time control devices highlights the significance of rigorous testing and validation in GIPVS research and development. In their study, Kulkarni et al. introduced a new current control system for inverters, aiming to effectively eliminate low-frequency harmonics. In order to address the issue of even harmonics in the generation of grid current, a proportional-resonant-integral controller approach was developed. This research looked at how the interaction between proportional-resonant-integral (PRI) controllers and the adaptive compensation system affected the overall system dynamics. The experimental findings validated the entire concept, exhibiting a strong correlation with the comprehensive theoretical analysis^62^. Kumar et al. conducted a comprehensive analysis of the difficulties associated with grid integration. Current and future developments in RES reliability and technology were discussed. Researchers identified a wide range of issues related to PQ, which were categorized, and a variety of solutions were investigated. Given the difficulties of RES integration into the grid, resolving them is essential if renewable energy adoption is to continue its ascent^63^. Grid-connected renewable energy systems can benefit from the use of FACTS devices, as investigated by Ali et al. The increased use of distributed generation (DG) in the grid has led to an increasing sensitivity of end-user devices towards PQ problems. Numerous studies pertaining to FACTS devices have demonstrated their effectiveness in mitigating PQDs, particularly in the context of integrating RES such as solar and wind into the power grid^64^. Thangaraj et al. introduced a novel approach for the regulation of solar power grids. To optimize power generation, the researchers conducted an investigation into the utilisation of MPPT in combination with a solar power system and a DC/DC converter for the purpose of sustaining a consistent DC-link voltage. In order to enhance the PQ of the grid-connected system, an active filtering technique was employed. The effectiveness of their approach was evaluated by dynamic simulations conducted using MATLAB or Simulink^65^.

Power electronics circuit reliability assessment in GCPVS was the focus of research by Priyadarshini et al. There were three different DC–DC converter topologies investigated for their reliability and mean time between failures (MTBF) when used for MPPT in GCPVS. They determined reliability and MTBF by computing the failure rate of each converter component, which yielded important insights into converter performance. All studies were performed in Python^66^. The integration difficulties of high-penetration PV systems of the future, which are projected to be more efficient and reliable, were studied by Yang et al. They investigated the feasibility of incorporating flexible power controllers into PV inverters and found that such controllers could be adapted to meet various requirements. Their 1-phase PQ model-based control technique established a basis for providing useful references for the current control's inner loop. This method was conditional on the existing infrastructure and the particular needs of the system's operators and end users^67^. For nonlinear loads, Yekanth et al. suggested a plan for GCSPV generators. To address VAR power and harmonic difficulties, they implemented a shunt active power filter (APF) with droop control. Using power references, the suggested controller showed theoretical improvements and provided a simple control topology. The PV component was wired into the Shunt APF's DC side through a DC–DC converter, with control provided by the Perturb and Observe (P&O) Maximum Power Point Tracking algorithm. The benefits of this system were verified by simulations in MATLAB Simulink^68^. In order to evaluate the efficiency of crystalline PV systems, Yunus et al. conducted a study using the MathCAD program. They used mathematical techniques to verify certain hypotheses about the operation of GCPV systems by comparing simulated results with computed data. The research set out to answer pressing questions about the general implementation of PV systems, such as the technology needs for GCPVS and the answers to harmonic problems^69^. Bai et al. introduce a fault ride-through method for grid-tied PV systems, enhancing their performance during voltage sags and mitigating inverter current during symmetrical faults. A constant active current reactive power injection technique optimizes LVRT operation for solar PV inverters in low-voltage grids. This approach effectively manages active and reactive power references, meets grid code standards, and eliminates tripping due to overcurrent. It enhances power converter responsiveness during voltage sags, providing improved dynamic grid support^70^.

Echeverria et al. examined many challenges related to PQ in the context of decentralized power generation systems. In the set-up of a novel large-scale photovoltaic plant design, the researchers conducted an investigation on distinctive power devices such as the STATCOM, UPQC, and DVC. The integration of the DC–DC stage interface was incorporated into the overall design of their modular multilevel converter-based HVDC system. The utilisation of PV systems' inherent DC characteristics offers advantages when integrated with HVDC networks^71^. The investigation conducted by Rashid al-Badwawi and colleagues focused on hybrid renewable energy systems. Voltage and frequency fluctuations, as well as harmonics, have emerged as significant PQDs, particularly in grid-connected and independent systems. These issues have a more apparent influence on weak grids. In order to effectively address these issues, the research highlighted the importance of meticulous planning, advanced rapid response management services, and the optimization of hybrid systems. The study conducted an extensive review of the existing literature related to optimal size designs, power electronics topologies, and control mechanisms^72^ in order to provide an in-depth understanding of the current state of research in this domain. Shahbaz Hussain et al. presented an HRES with a modular design as a means to attain cost-effectiveness and high dependability in project implementation. A significant objective of the design was to minimize energy waste. The designers of the hybrid PV-Wind Turbine Battery System used an iterative filter selection method to find the best possible design among many possible options. The study employed the iterative Pareto-fuzzy method to assess the performance and cost-effectiveness of the system. The objective was to enhance project cost efficiency and meet capacity requirements^73^. Many researchers have introduced the cascaded H-Bridge DVR-interfaced PV system to enhance the PQ. A PV-integrated DVR using a rotating dq reference frame controller with an optimum tuned PI controller is proposed to minimize voltage sag and swell while preserving DC link voltage for loads and improving PQ. An ANFIS algorithm is used to tune the reference frame-based PI controller of the DVR^74^. In reference^75^, the implementation of a DVR is utilised to resolve the problem of an asymmetrical fault inside a grid-connected system. This system operates within a 3-phase grid-connected configuration and aims to mitigate PQDs that occur from the PV-grid side, particularly during adverse weather conditions. One approach to improving PQ is through the implementation of active power filters. It emphasizes performance, implementation, and THD measurement. The DVR controller stands out for its fast response, offering a cost-effective and reliable solution to maintain consistent performance, particularly in mitigating harmonics and voltage fluctuations^76^. To improve the main grid terminal PQ, a multifunctional grid-connected voltage-source inverter (MFGCVSI) was in charge of controlling solar PV active power injection^77^. Adjustable DC-link voltage regulation could reduce inverter switch load and switching losses. The implementation of simulations and hardware testing resulted in a decrease in the average switching frequency and an enhancement in the balance of split capacitor voltage, hence leading to a reduction in switching losses. The authors of^78^ have presented an adaptive control system for GIPVS. The system has several advantageous features, including unity power factor, effective harmonic suppression, efficient management of the dc-link voltage, and seamless mode changes. The attainment of power management and the extraction of power under varied conditions are effectively accomplished via the utilisation of a bidirectional converter and battery unit. A new current reference control technique improves power tracking, and a power management algorithm optimizes operation. For problems with PQ posed by non-linear loads, this study presents a method of controlling photovoltaic converters that are incorporated into the grid. The technique employed by the inverter leads to increases in power factor, reactive power management, and harmonic reduction. The grid-connected inverter is transformed into a shunt-connected active filter at the point of common coupling through the process of separating the fundamental active load current component in order to provide a compensation signal. This action serves to improve the power quality of the utility grid^79^. According to the findings of the study conducted by researchers^80^, suggests a two-stage, 1-phase PV grid connection using a high-frequency transformer. The first stage uses a buck-boost inverter coupled with energy storage to improve MPPT and deal with PV voltage changes. Power quality is improved, and leakage current is decreased with high-frequency transformers. Second stage power density, grid filter reduction, and reliability are achieved by high switching frequency interactions between a rectifier-inverter system and the utility grid. In^81^, a new control technique for multifunctional grid-connected photovoltaic systems (GCPVSs). While considering inverter capacity, it prioritizes PCC power quality enhancement. Reduce dc-link voltage oscillation with an Adaptive Neuro-Fuzzy Inference System (ANFIS)-based MPPT controller for a two-phase interleaved boost converter. It handles active power injection, reactive power adjustment, and harmonic filtering, focusing on the former. The approach is tested in MATLAB/Simulink with different solar irradiation levels in a grid-connected PV system. Recent research^82^ shows that MPPT algorithm sample rate and step size affect PV inverter inter-harmonics emissions. Faster rates and greater steps improve tracking but hurt inter-harmonics and efficiency. The paper examines inter-harmonics in operating contexts and recommends mitigation. Simulations show that MPPT parameter changes lower inter-harmonics by 27%, improving PQ without affecting performance. The regularized least logarithmic absolute difference (RLLAD) filter is introduced to limit harmonic generation in VSC output current, enhancing utility grid power quality for nonlinear/linear loads. IEEE standards say the adaptive RLLAD filter balances sinusoidal grid currents. The MATLAB/Simulink simulations shown in^83^ showcase the concepts of harmonic filtering and transient response. In summary, the exponential expansion of grid-connected photovoltaic systems (GIPVS) presents a number of technological and economic challenges. The factors discussed in this context include the strain exerted on the utility grid during the shift from peak to off-peak generation, the impact of peak shaving, and the inherent unpredictability associated with GIPVS installations. Researchers have suggested a variety of ways to address these difficulties, including transformer-less inverters, solar tracking, and maximum power point tracking (MPPT) methodologies. Inverters play a crucial role in providing ancillary services like VAR power management and frequency regulation. Moreover, advanced control strategies, energy storage, and grid integration methods have been explored to enhance the performance and stability of GIPVS. Despite these advancements, power quality disturbances, such as voltage fluctuations and harmonics, remain a significant concern. A range of control mechanisms and devices, such as dynamic voltage restorers (DVR), active power filters (APF), and shunt active power filters, have been created by researchers to address PQ difficulties. Additionally, the integration of GIPVS into the grid has prompted investigations into improved grid management techniques. These efforts involve the use of flexible power controllers, FACTS devices, and advanced control systems to maintain PQ and reliability. Furthermore, researchers have explored the use of supercapacitors and novel current control schemes to enhance PQ and system reliability in GIPVS. The findings from these studies highlight the importance of rigorous testing and validation using simulation tools like MATLAB/Simulink and real-time control devices. Overall, addressing the challenges associated with GIPVS integration into the grid requires a multi-faceted approach involving advancements in technology, control strategies, and grid management practices. The comprehensive review of Challenges and Trends of grid integrated wind energy system discusses in this paper along with advantages are summarized in Table 1.

**Table 1:** Resent advancements and emerging trends in grid connected wind and solar system.

S. no.	Authors	References	Advantages	Issues addressed
1	Muhammad Abdul Basit et al	^16^	Mitigated the current harmonics with APF Converter dynamic profile used in RESs can be handled with VIC. VIC furthermore provided regulation of power flow, stability, and unbalance compensation	Instability, flickers in voltage, fluctuation in voltage, and cascaded fault events
2	Ashok Kumar and Indragandhi	^17^	Provided VAR support, good harmonic mitigation as well as preserving the source current and voltage not including phase angle variation	Power quality improvement
3	Ayodele et al	^18^	Get better the flexibility in addition to raise the combination of wind power to grid	Influence of wind power on system security, Wind power impact on power quality (harmonics, flickers, voltage dip), challenges of wind power on stability of power system, and mitigation strategies for WECS integration
4	Shakir D. Ahmed et al	^19^	This article reviewed existing solution methods together with grid codes, energy storage schemes, and wind energy strategy to struggle with issues	It focused on wind energy intermittency, VAR support, stability of voltage as well as frequency, PQ problems, fault ride-through ability, security, cyber Security, electrical energy market, planning, socio-economic, along with environmental challenges
5	Thabeti Aditya et al	^20^	The feasible solutions for the PQ issues, technological issues in GIPV scheme for the trust worthy function of the system were suggested	Swell in voltage, sag in voltage, imbalance in voltage, problems of flickering, harmonics in current and voltage, issues related to power factor, power reverse flow, regulation of voltage, variation of VAR, variation in frequency that were linked to grid connected PV system. Certain key technological challenges included short as well as long term power interruption, protection, storage, islanding, reliability; stability associated issues were also mentioned in this study
6	Abdel-Raheem Youssef et al	^21^	Improved efficiency and fast system response. High efficacy and simple in working	Overall system efficiency
7	Jawad Hussain et al	^22^	DSTATCOM with BESS can be effectively utilized to enhance the PQ of wind power distribution networks	PQ problems like voltage swells, voltage dips, harmonics, PF and voltage regulation
8	Jayanthi et al	^23^	Mitigated the high fault current when there is a fault in the grid	Grid voltage and current during faults
9	Panigrahi et al	^24^	Provided a solution to wind power modeling in addition to PV source modeling and its balancing to grid	Impact of wind flow on voltage and VAR and active power
10	Srinivas et al	^25^	Various MPPT techniques along with its comparison is carried	–
11	Loo Choon Phei et al	^26^	MC controlled the terminal voltage in addition to frequency of the synchronous generator	Terminal voltage and frequency
12	Ndirangu J.G. et al	^27^	–	Voltage fluctuations, Harmonics, frequency fluctuations, virtual inertia
13	Amita et al	^28^	Improved PQ in system	Security, reliability, availability and quality associated to power to be supplied, either to grid or to load center
14	Daryabi et al	^29^	It showed an outstanding performance in maintaining the PQ profile as per requirement	Power quality
15	Magesh et al	^30^	Golden Eagle Optimisation Algorithm (GEOA) controllers Handle the nonlinearity of systems well, so they can be Used in microgrids, smart grids, charging stations for electric vehicles, and energy storage systems for green energy. Maintaining the PQ profile as per requirement	Improve the performance of grid-connected Permanent Magnet Synchronous Generator driven by Variable-Speed Wind Turbine
16	Rawa M et al	^31^	Covers two different modes of operation, off grid and grid Linked, both with significant nonlinearloads	When confronted to voltage disturbances from the grid It responded well and rejected harmonics Without sacrificing voltage quality
18	M. Feyzi et al	^32^	Enhance the capability of the LVRT Better and quicker dynamic reaction under both unbalanced and balanced settings Inject active and reactive power	Control of DC-link voltage, active and reactive power injection under balanced and unbalanced condition
19	Naderi et al	^33^	Reduce the effect of the defect more quickly by improving the damping performance and reducing the overshoot	Power quality during symmetrical and asymmetrical faults
20	J. Li et al	^34^	Reduce the variability of dc voltage as well as the cost of the equipment	DC bus voltage fluctuations
21	M. M. Islam et al	^35^	Reduce the fault currents in an effective manner; prevent a drop in voltage	Fluctuations in voltage and current
22	Song, Yuyan, et al	^36^	Reduce the rotor overcurrent in an effective manner with the help of the Q-learning based robust model predictive control method	DFIG rotor overcurrent
23	R. Hiremath and T. Moger	^37^	Improved LVRT capabilities Effectively manages severe grid fault system, overall harmonics distortion and Improved LVRT capabilities Effective in managing system uncertainties from major grid faults	Harmonic distortion and grid faults
24	Gupta S et al	^38^	Transients are handled well by VSC control. The controller improves power quality and compensates for reactive power. Unity power factor and uninterruptible power supply to the nonlinear load are also provided	Improving the quality of power, compensating for the reactive power and unity power factor
25	Watil Aziz et al	^40^	Tackles the issue of operating a self-contained wind energy conversion system by utilising battery storage for energy	Power quality challenges
26	Krishna, D et al.	^41^	FOFL controller-based UPQC addresses power quality issues including harmonics, voltage sag/swell, and VDC-Link regulation, enhancing system dynamics. Its effectiveness is demonstrated in a nonlinear load power distribution system	Current distortion, voltage Swell, Sag, and THD of nonlinear loads in distribution power system
27	Krishna et al.	^42^	Improve the power by utilising an Adaptive FLC-based UPQC system	Harmonics, voltage sag and swell
28	Ahilan, T	^43^	In order to enhance the power quality of the system, we made use of a total of four distinct types of compensators, including UPFC, DPFC, USSC, and UPQC	Problems with the power quality include swell, interruptions, sag, and harmonics
29	Ismail, M.M. and Bendary, A.F	^46^	Enhancing battery life and reliability by grid connection based on state-of-charge and sun irradiation percentages	Designed a smart controller to maximize battery use and lifespan
30	Chakir, A. et	^47^	When the load is powered, an optimal algorithm handles energy management and source synchronization	Examine the Contentment of Electricity Consumers
31	Bhuwan Pratap Singh et al	^48^	Enhanced the performance of the grid connected PVS	Impacts on voltage regulation devices, equipment loading and power losses, Harmonics, VAR regulation, active power regulation, frequency stability, voltage stability
32	Ahmed Jumui Sumoi Fomba et al	^49^	Provided a possible design about solar energy generation potential at specified location	High-quality output power and system stability
33	Hicham Bahri et al	^51^	It reduced system cost and instability of the controller by removal of measurement noise	System cost and instability of the controller
34	Najib Hamrouni et al	^52^	High performances in transient and permanent phases	Reliability in the grid fault process and to evade catastrophic breakdown
35	I.Hamdan et al	^53^	The simulation outcomes presented an obvious enhancement for the system stability with SC	Stability
36	Selmi T and Gastli A	^54^	CPDs are established to be very capable in combining solar and wind energy sources to the grid. They showed a significant part in the conception of CPD in carrying quality power at different levels	Quality of power
37	Walaa Ibrahim Gabr et al	^55^	Significant enhancement of PQ	Impact of GIPVS on THD
38	Rakeshwri Pal et al	^56^	Enhance the power quality of the system	PQ issues like voltage sag and voltage swell
39	Soumyadeep Ray et al	^58^	It is useful and edifying to the engineers, investigators, and industries	Efficiency increment, Low cost, and robustness of the whole scheme
40	Abhijit Kulkarni et	^62^	Good enhancement in the grid current THD	Lower order harmonics into the grid due to high-frequency PWM
41	Dr. S.M. Ali et	^64^	Very capable in combining solar wind energy sources to grid	Power quality
42	Kokilavani Thangaraj et al	^65^	It removed the need for extra power conditioning equipment to enhance the PQ	PQ issues like harmonics, voltage regulation etc.
43	Priyadarshini. K, and A. B. Raju	^66^	By comparing the reliability of three DC–DC converter topologies it is evident that among step-down converters Buck-boost is more reliable and among step-up converters Boost converter is more reliable	Reliability
44	Yekanth K et al	^68^	Shunt active power filter with droop control satisfies the reactive power requirement and reduces harmonics in the system	VAR power and harmonic difficulties
45	Bai K et al	^70^	Fault ride-through can improve grid-connected PV system performance and reliability, especially in low-voltage grids	response during voltage sags and limiting the maximum inverter current during symmetrical faults
46	Devalraju, and C.Dhanamjayulu	^74^	Effectively reduces voltage sag and swell, maintains load DC link voltage, and improves power quality	voltage sag and swell, maintain load DC link voltage
47	Farooqi, Awais, et al	^75^	Power quality difficulties in PV-grid systems during thunderstorms and high winds must be addressed to minimize transmission network instability from line-to-ground, lightning strikes, and power outages	Voltage overshoots, transient response, and steady-state errors cause microgrid instability during unsymmetrical distribution network outages and harm sensitive loads
48	Kumar, C, et al	^76^	Effectively mitigate the power quality issues using DVR based controller	Voltage sag, voltage swell and harmonics
49	Karasala, C. and Ganjikunta, S.K	^77^	Enhancing power quality at main grid terminals by regulating active power injection from the SPV system. Adaptive dc-link voltage regulation reduces inverter switch load and switching losses	DC-link voltage regulation, switching losses and burden on inverter switches
50	Priyanka Kishor Sorte et al	^78^	Maximum power extraction, quicker dc-link voltage management, smooth transition between modes of operation, active power feeding to the grid/loads, and excellent PCC power quality were obtained	D-link voltage regulation, smooth transfer of mode switching, harmonic mitigation and unity power factor operation
51	Surasmi NL and shiny G	^79^	Reduce power quality difficulties caused by non-linear loads	Reactive burden control, harmonic distortions, and power flow control and improved power factor
52	Rashwan, Ahmed, et al	^80^	Employed two-stage grid-connected inverter topology with high frequency link transformer to improve power quality	MPPT during large PV voltage changes and power quality of the system
53	Soumana, R.A et al	^81^	The PV inverter's control technique ensures simultaneous active power injection, reactive power correction, and current harmonic filtering	DC-link voltage oscillation, current harmonic filtering and reactive power compensation
54	Hussein, I. et al	^82^	MPPT efficiency is investigated by minimizing inter-harmonics emission and operating dynamically	Investigates inter-harmonics production and emission at different power conditions
55	Patel N et al	^83^	Enhance power quality by managing VSC output current harmonics in utility grid connections with nonlinear/linear loads	Grid current balancing and harmonic suppression, active and reactive power management, neutral current compensation, and power factor correction
56	Choube and S H	^84^	The wind turbine’s performance and PQ are determined. By measurements and standards followed according to The IEC-61400 standard	Improves Power quality
57	Soumyadeep Ray et al	^85^	It is useful and edifying to the engineers, investigators, and industries	Efficiency increment, Low cost, and robustness of the whole scheme

Following an examination of a number of different pieces of published research, the research gap in power quality disturbances in grid-connected wind and solar systems can be summarized as follows.

- *Integration of Renewable Resources*: As the integration of wind and solar power into the grid continues to expand, exhaustive studies on the unique power quality challenges and disturbances that result from the intermittent and variable nature of these renewable resources are necessary. The focus of research should be the development of effective solutions for mitigating disturbances induced by the variable nature of renewable energy production.
- *Harmonic Distortion*: Harmonic distortion issues relating to grid-connected wind and solar systems must be investigated further. Harmonics can negatively impact the performance of sensitive equipment and increase grid losses. To reduce harmonic content, research should strive to develop advanced filtering and control techniques.
- *Voltage Fluctuations*: Voltage fluctuations, such as voltage dips and surges, can have a significant effect on the stability and dependability of grid-connected renewable energy systems. The focus of research should be on refining voltage regulation and the ability of these systems to withstand voltage disturbances.
- *Grid Interconnection Standards*: To ensure consistent power quality and system performance, grid-connected wind and solar systems require standardized guidelines and regulations. The focus of research should be on devising and revising standards that take into account the specific characteristics of renewable energy sources.
- *Integration of Energy Storage*: The integration of energy storage systems (e.g., batteries) with grid-connected renewable energy systems can mitigate power quality disturbances. To enhance overall system performance, research should investigate optimal sizing, control strategies, and coordination between renewable sources and energy storage.
- *Advanced Control Algorithms*: In grid-connected systems, it is essential to develop advanced control algorithms tailored to the specific requirements of LVDT, DVR, STATCOM, APF, UPFE, and UPQC. To provide rapid and precise responses to a variety of power quality issues, research should address the difficulties of controlling these devices.
- *Hybrid Systems*: Examine the potential advantages of hybrid systems that combine multiple custom power devices to address a wider variety of power quality disturbances. Research should investigate the synergies and costs of integrating these devices into a single system.
- *Cost-Effectiveness*: Evaluate the cost-effectiveness of deploying custom power devices in renewable energy systems that are grid-connected. Analyze the economic viability of these devices in terms of their installation, upkeep, and overall impact on the enhancement of power quality.
- *Standardisation and Interoperability*: Establish standardised deployment and interoperability guidelines and protocols for custom power devices in grid-connected systems. Ensure that these devices are compliant with industry standards and are compatible with renewable energy installations.
- *Modelling and Simulation*: Enhance the modelling and simulation tools used for the design, analysis, and optimisation of the performance of custom power devices in grid-connected wind and solar systems. This includes creating accurate simulations of these devices operating under a variety of conditions.

## Proposed power quality mitigation technique

Based^84^ on the literature review above, it is clear that power quality disturbances (PQDs) happen when nonlinear loads are added to the power distribution system and when solar and wind power are added. Multi-feeder interline unified power-quality conditioners (MF-IUPQCs) utilise fuzzy logic controllers (FLCs) to address power quality disturbances (PQDs), including voltage and current fluctuations as well as harmonic distortions. This section provides a description of the application of FLCs in MF-IUPQCs for this purpose. The MF-IUPQC consists of three legs and three levels, with each level consisting of four diode-clamped inverters. Switching is carried out through the use of space vector pulse width/duration modulation (SVPWM). The FLC-based MF-IUPQC is effective in reducing total harmonic distortion (THD) caused by nonlinear loads. Additionally, it enhances dynamic performance and provides a stable DC-link voltage. The proposed control mechanism is implemented using MATLAB and Simulink. The effectiveness of the fuzzy-based controller is evaluated by comparing it to the industry-standard proportional-integral (PI) controller. With regard to both THD and voltage profile smoothness, the MF-IUPQC based on FLC performs best. The unified power quality conditioner (UPQC) is a specialized power device that corrects PQDs in the distribution network caused by voltage and current^86^. The UPQC perform shunt and series combination to solve many PQDs. Both the DVR and DSTATCOM compensators improve the current and voltage profiles, respectively. However, numerous multi-feeder CPDs such as an interline voltage controller (IVOLCON), an interline dynamic voltage restorer (IDVR), an interline power-flow controller (IPFC), and an IUPQC with dual VSCs have been reported in the literature^87^. These multi-feeder CPDs operate on the principle of using power from a neighboring healthy feeder to offset the deficiencies in the existing local feeder. PI controllers are more common due to their simple design and operation. However, adapting to complicated nonlinear systems is difficult. In^88^ Jindal et al., proposed IUPQC with two VSCs, in this the shunt connected converter improves current profile in one feeder whereas the series connected converter improves voltage profile in second feeder. Switching action of each VSC is performed independently by using pole-shift controller.

### System configuration

#### HES system

**Figure 5:**
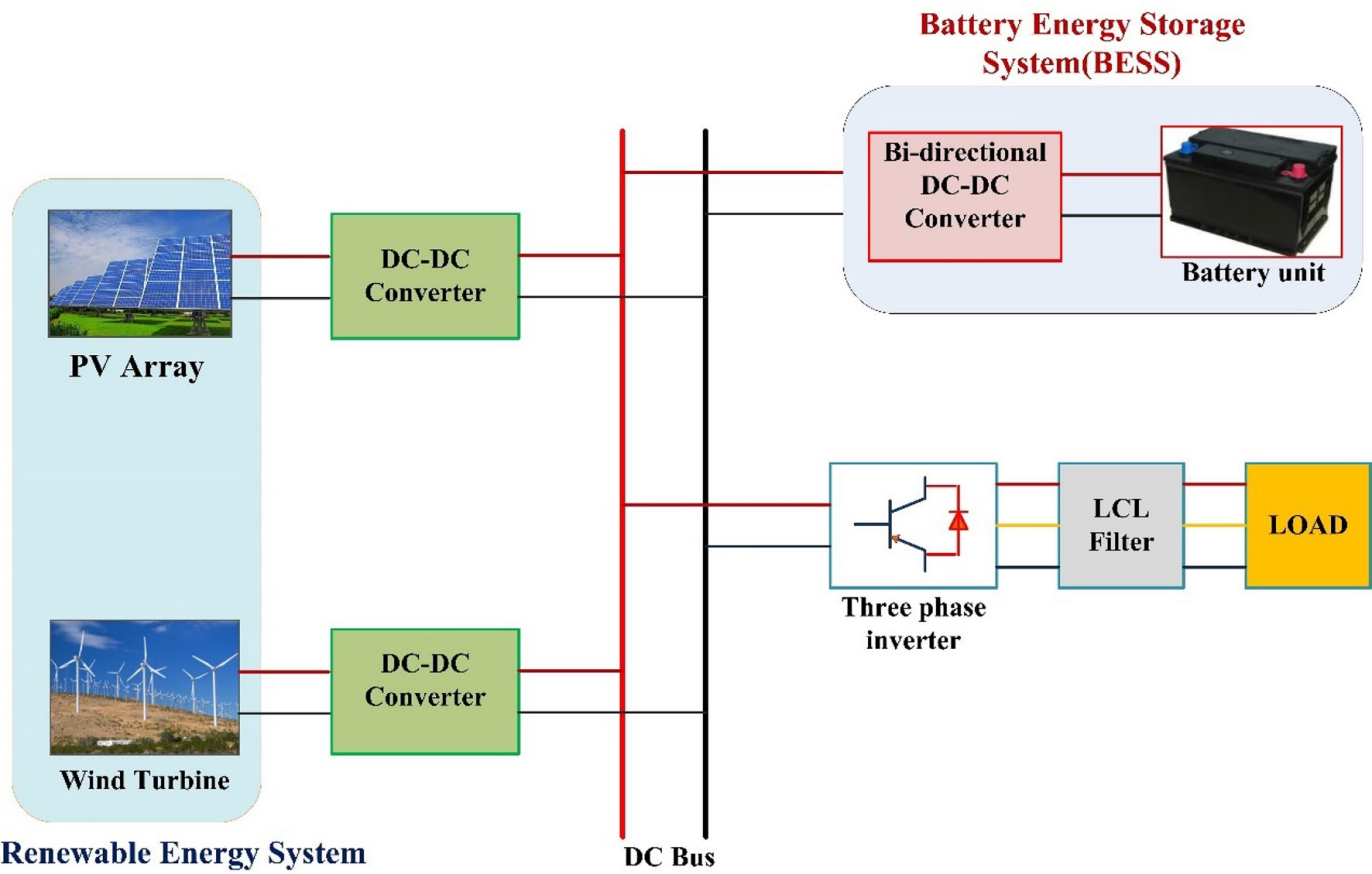
HES system.

**Figure 6:**
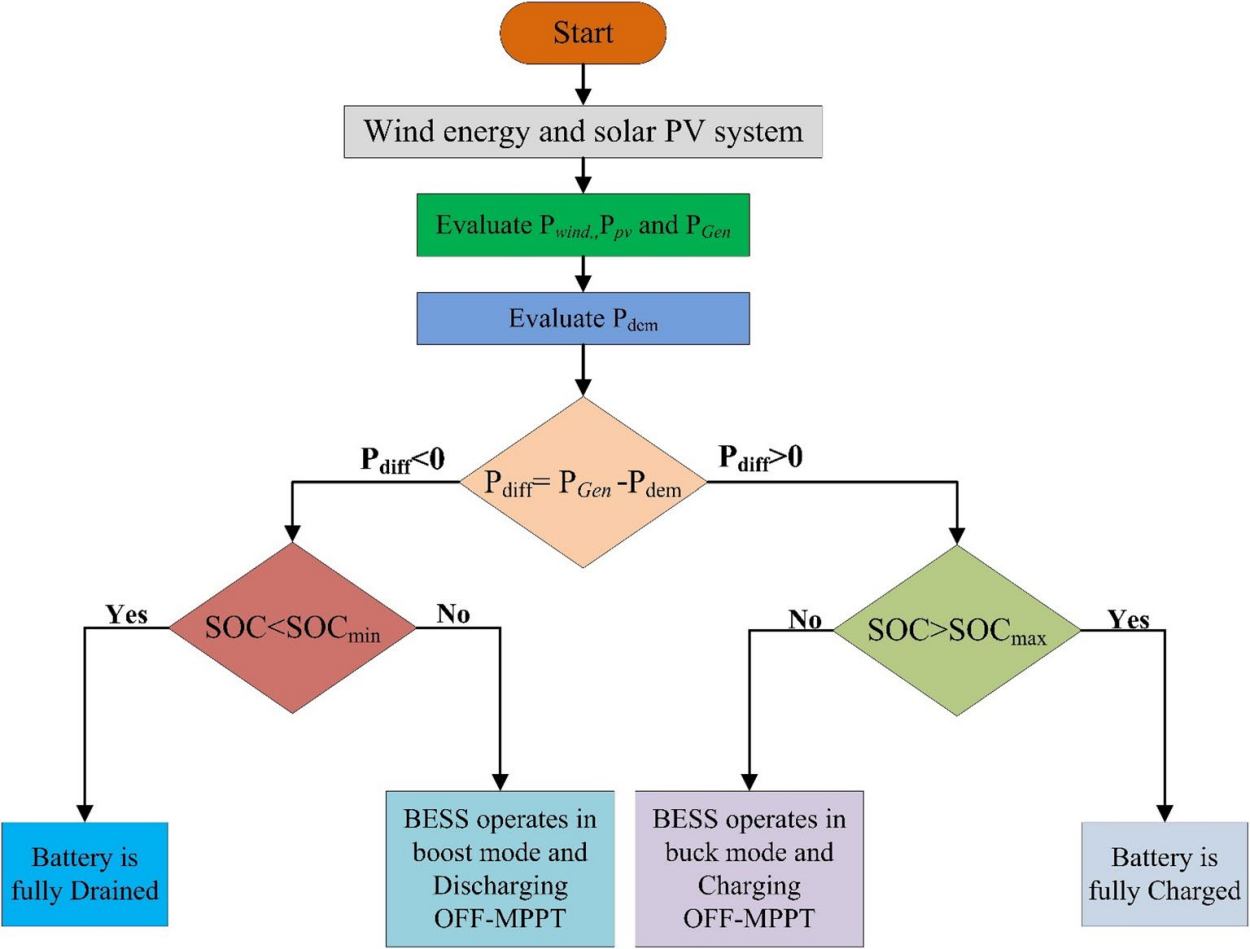
Flow chart.

Basic HES system is shown in Fig. 5. The major equipments of the system are permanent magnet synchronous generator (PMSG) based wind turbine, PV module, rectifier, and DC–DC converter. The working of HES is depicted in Fig. 6 using flowchart.

#### Photovoltaic array

The PV cell V-I characteristics are non-linear and vary with solar irradiance and temperature. The DC–DC boost converter receives the output of the photovoltaic array. The boost converter's output is connected to the DC bus. The PV array generates 646 V of power, which is then increased to 1000 V via a boost converter. The Eqs. (1) and (2) gives the diode and load currents of the PV module as follows:

1 $${I}_{d}={I}_{sat}\left({e}^{\frac{Q{V}_{oc}}{AkT }}-1\right)$$

2 $$I={I}_{L}-{I}_{sat}\left({e}^{\frac{Q{V}_{oc}}{AkT }}-1\right)-\frac{{V}_{oc}}{{R}_{sh}}$$

3 $${V}_{oc}=\frac{AkT}{Q }{\mathrm{log}}_{n}\left(\frac{{I}_{L}}{{I}_{sat}}+1\right)$$

#### Wind energy

The PMSG based wind turbine converts the wind kinetic energy into mechanical energy. The output of PMSG is fed to uncontrolled bridge rectifier. The DC–DC boost converter receives the output of the rectifier. The boost converter's output is connected to the DC bus. Power captured by the wind turbine is as follows (Eq. (4)):

4 $$P=\frac{1}{2}\rho A{v}^{3}{C}_{p}\left(\uplambda ,\upbeta \right)$$

where $P$ is generated power, ρ is density of air, A is the blades swept area, v is the velocity of wind, β is the pitch angle and λ is the tip-speed ratio (TSR), Cp is the power coefficient which depends on λ and β can be formulated as:

5 $${C}_{p}\left(\lambda ,\beta \right)=0.5176\left(116\times \frac{1}{{\lambda }_{1}}-0.4\beta -5\right){e}^{-\frac{21}{{\lambda }_{i}} }+0.068\lambda$$

where

6 $$\frac{1}{{\lambda }_{i}}=\frac{1}{\left(\lambda +0.08\beta \right)-(\frac{0.035}{1+{\beta }^{3}})}$$

The torque is given as:

7 $$T=\frac{P}{{\omega }_{m}}$$

where ${\omega }_{m}$ is rotor speed. As seen in Eq. (7), for each wind speed, there is an optimal rotor speed at which maximum power is achieved.

#### Multi-feeder interline unified power quality conditioner (MF-IUPQC)

**Figure 7:**
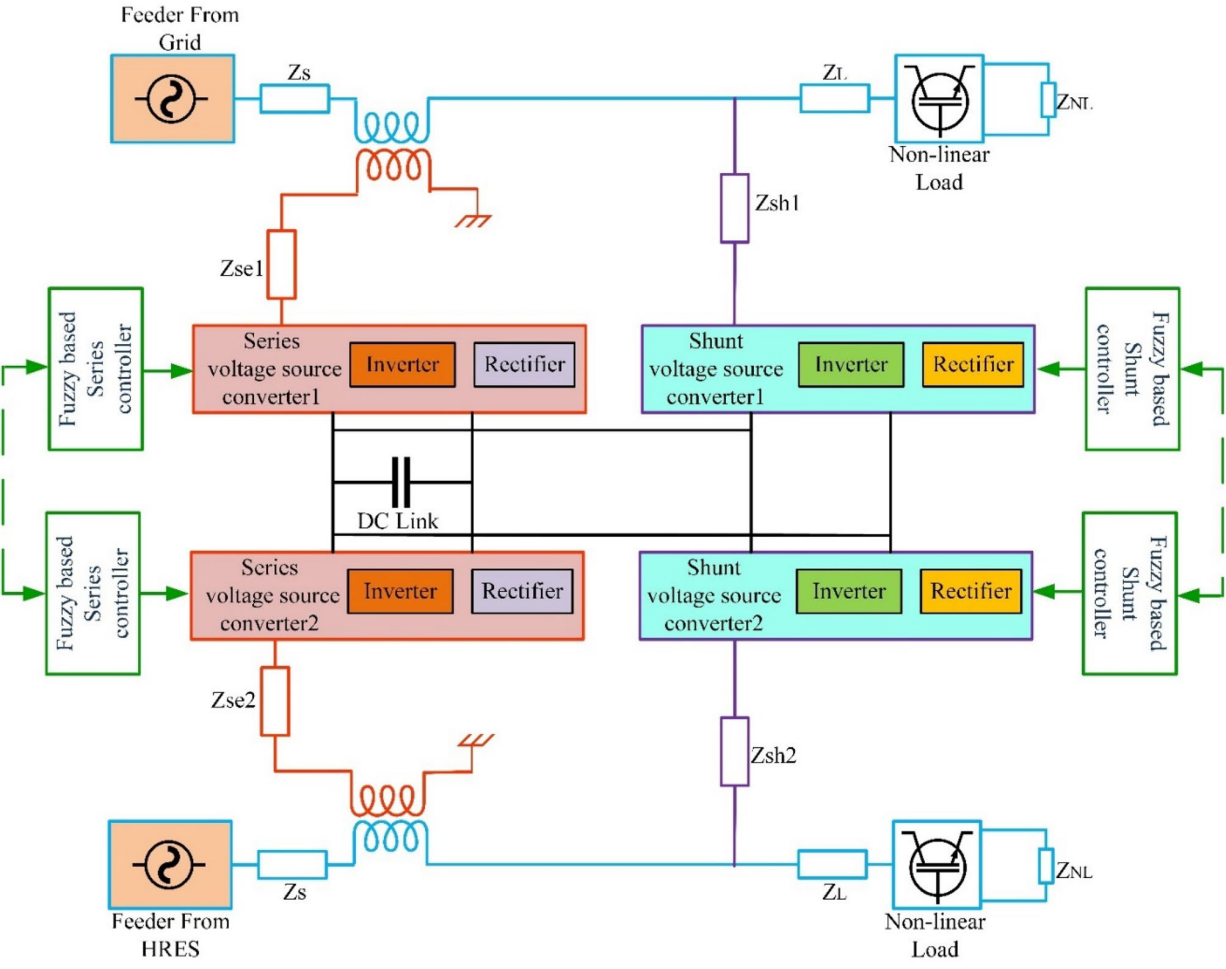
Schematic diagram of MF-IUPQC.

The MF-IUPQC is created by connecting two series VSCs and two shunt VSCs back-to-back via a common DC-link capacitor, as demonstrated in Fig. 7 This creates the MF-IUPQC. In this instance, each VSC's control is handled separately. The following sections discuss the operation of the controllers.

### Control method

#### Series controller

**Figure 8:**
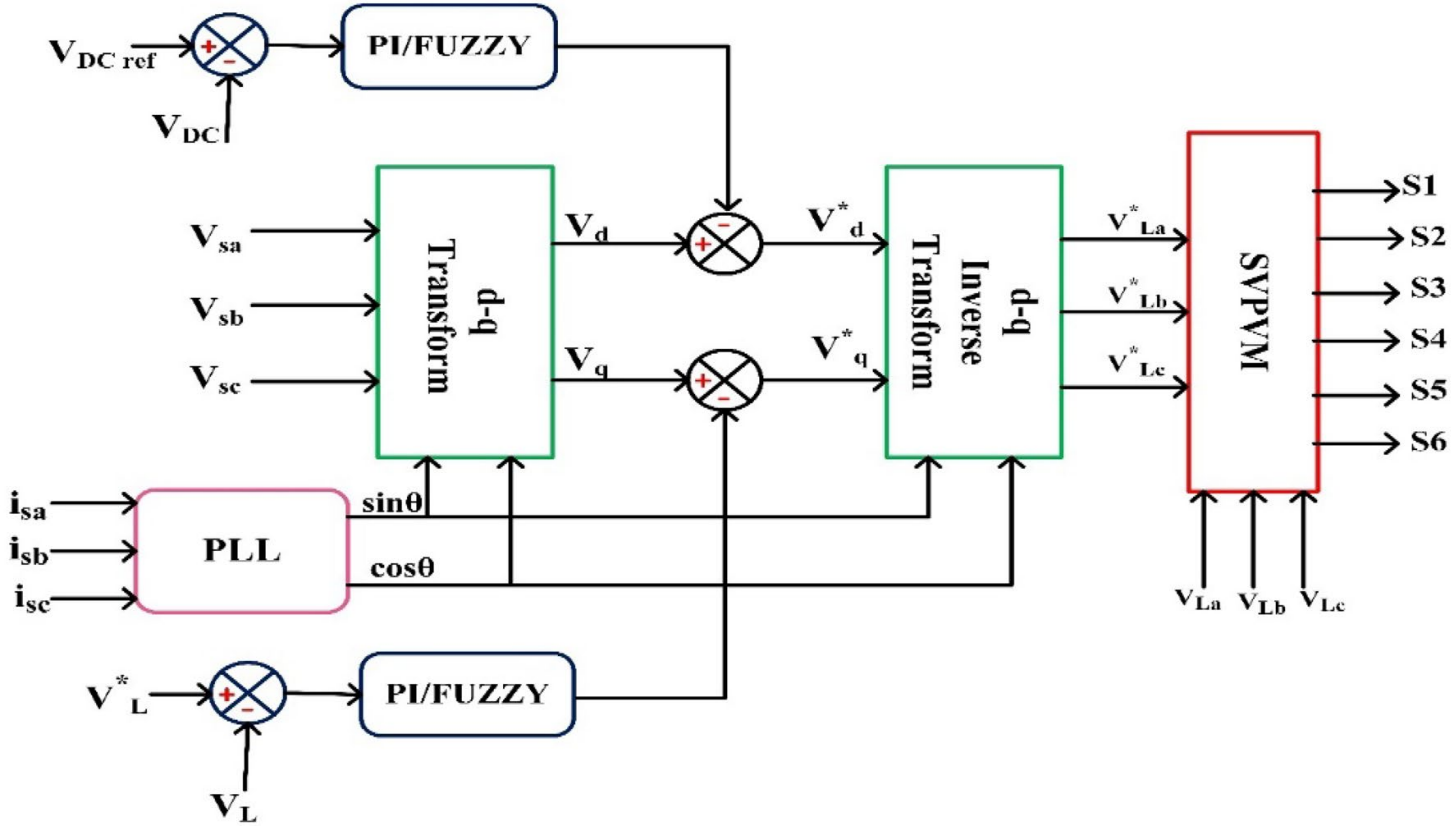
Schematic diagram of series control.

Figure 8 provides an illustration of the synchronous reference frame (SRF) theory, which is applied in order to manage the series VSC for each feeder. At this point, Park's transformation is applied to the three-phase source voltage ${V}_{sabc}$ in order to produce the rotating reference frame ${V}_{sdq}$ using. Equation (8) provides a description of this phenomenon.

8 $$\left[\begin{array}{c}{V}_{sd}\\ {V}_{sq}\end{array}\right]=\frac{2}{3}\left[\begin{array}{ccc}\mathrm{sin}\omega t& \mathrm{sin}\left(\omega t-\frac{2\pi }{3}\right)& \mathrm{sin}\left(\omega t+\frac{2\pi }{3}\right)\\ \mathrm{cos}\omega t& \mathrm{cos}\left(\omega t-\frac{2\pi }{3}\right)& \mathrm{cos}\left(\omega t+\frac{2\pi }{3}\right)\end{array}\right]\left[\begin{array}{c}{V}_{sa}\\ {V}_{sb}\\ {V}_{sc}\end{array}\right]$$

The d-axis elements are often used to eliminate harmonics and compensate for reactive power. The output of a rotating reference frame is dependent on the source voltages and the phase lock loop (PLL) output. The output voltage like (${V}_{dq}$) is compared to reference voltages like (${V}_{dqref}$) after the transformation process. When the actual and reference voltage vectors are compared, some error quantities are obtained. The PI/Fuzzy controller minimizes these error quantities. In the end, the mistake is corrected, and the results are used to generate the reference voltage in the d-q frame as (${V}_{dq}^{*}$). With the use of the inverse transformation process, the reference voltage values from the $d-q$ frame can be re-converted into the $abc$ frame. This procedure is outlined in Eqs. (9) and (10).

9 $${V}_{dq}^{*}={V}_{dqref}-{V}_{dq}$$

10 $$\left[\begin{array}{c}{V}_{La}^{*}\\ {V}_{Lb}^{*}\\ {V}_{Lc}^{*}\end{array}\right]=\frac{2}{3}\left[\begin{array}{ccc}\mathrm{cos}\omega t& \mathrm{sin}\omega t& 1\\ \mathrm{cos}\left(\omega t-\frac{2\pi }{3}\right)& \mathrm{sin}\left(\omega t-\frac{2\pi }{3}\right)& 1\\ \mathrm{cos}\left(\omega t+\frac{2\pi }{3}\right)& \mathrm{sin}\left(\omega t+\frac{2\pi }{3}\right)& 1\end{array}\right]\left[\begin{array}{c}{V}_{d}^{*}\\ {V}_{q}^{*}\end{array}\right]$$

The switching pulses are generated by comparing the voltages of the reference (${V}_{Labc}^{*}$) and load (${V}_{Labc}$) voltages, and then sending those comparison results through the SVPWM controller.

#### Shunt controller

**Figure 9:**
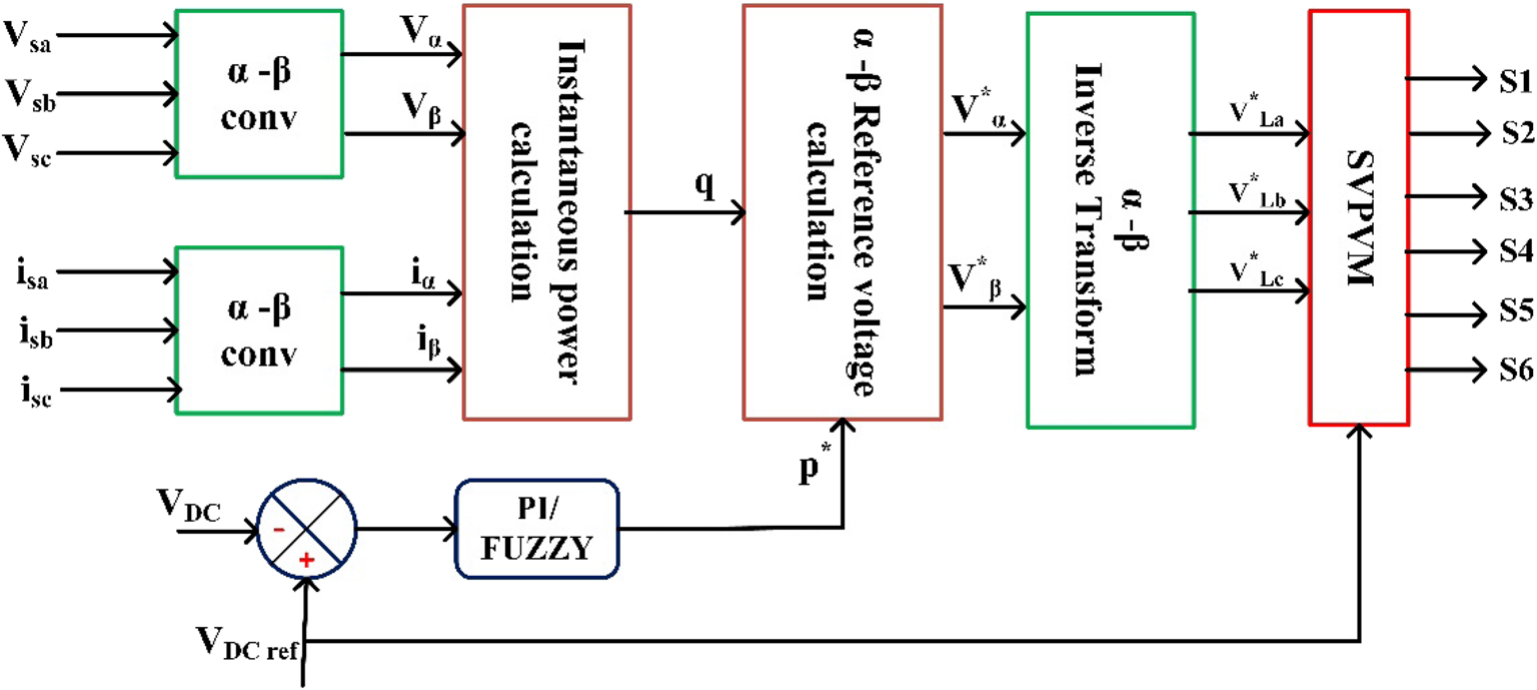
Schematic diagram of Shunt control.

Figure 9 provides an illustration of the Instantaneous active-reactive power theory, which is applied in order to adjust the shunt VSC for each feeder. In this step, the three phase load voltages and currents are recalculated using the $\alpha -\beta$ coordinate system. The explanation for this can be found in Eqs. (11) and (12).

11 $$\left[\begin{array}{c}{V}_{L\alpha } \\ {V}_{L\beta }\end{array}\right]=\sqrt{\frac{2}{3}} \left[\begin{array}{ccc}1& -\frac{1}{2}& -\frac{1}{2}\\ 0& \frac{\sqrt{3}}{2}& -\frac{\sqrt{3 }}{2}\end{array}\right]\left[\begin{array}{c}{V}_{La}\\ {V}_{Lb}\\ {V}_{Lc}\end{array}\right]$$

12 $$\left[\begin{array}{c}{I}_{L\alpha } \\ {I}_{L\beta }\end{array}\right]=\sqrt{\frac{2}{3}} \left[\begin{array}{ccc}1& -\frac{1}{2}& -\frac{1}{2}\\ 0& \frac{\sqrt{3}}{2}& -\frac{\sqrt{3 }}{2}\end{array}\right]\left[\begin{array}{c}{I}_{La}\\ {I}_{Lb}\\ {I}_{Lc}\end{array}\right]$$

Using the aforementioned equations, one can make an approximation of the instantaneous active and reactive components^89^.

13 $$\left[\begin{array}{c}p\\ q\end{array}\right]=\left[\begin{array}{cc}{V}_{L\alpha }& {V}_{L\beta }\\ -{V}_{L\beta }& {V}_{L\alpha }\end{array}\right]\left[\begin{array}{c}{I}_{L\alpha }\\ {I}_{L\beta }\end{array}\right]$$

The active and reactive powers in the previously mentioned Eq. (13) are able to have both mean and oscillatory components. The oscillatory component of load currents has a negative sequence component, whereas the mean component has a positive sequence component. Both components are described by their respective means. To maintain proper synchronization with the supply, the desired reference voltage values ${V}_{La}^{*}$, ${V}_{Lb}^{*}$ and ${V}_{Lc}^{*}$ (shown in Eq. (14)) are produced in conjunction with the power references. In^90^ addition to the DC reference voltage, the voltage references are transmitted to the SVPWM controller. This causes the controller to create the required number of switching signals for the shunt VSC switches.

14 $$\left[\begin{array}{c}{V}_{La}^{*}\\ {V}_{Lb}^{*}\\ {V}_{Lc}^{*}\end{array}\right]=\sqrt{\frac{2}{3}} \left[\begin{array}{ccc}\frac{1}{\sqrt{2} }& 1& 0\\ \frac{1}{\sqrt{2}}& -\frac{1}{\sqrt{2}}& \frac{\sqrt{3}}{2}\\ \frac{1}{\sqrt{2}}& -\frac{1}{\sqrt{2}}& -\frac{\sqrt{3}}{2}\end{array}\right]$$

The control strategy plays a crucial role in enhancing the efficiency of the Interline Unified Power Quality Conditioner (IUPQC) and mitigating power quality (PQ) issues such as voltage sag, swell, and harmonics. This paper proposes two potential solutions, namely PI and Fuzzy controllers, for addressing PQ problems in the IUPQC system. The control strategy of the controller follows a three-step process to handle PQ issues. Firstly, it should accurately determine the system voltage. Secondly, it generates the required switching pulses for the converter to operate effectively. Lastly, it generates the appropriate reference voltage for adjustment purposes. By implementing this control strategy, the IUPQC system can effectively regulate the voltage and compensate for PQ disturbances, resulting in improved power quality and reliable operation of the electrical grid.

#### Fuzzy controller

**Figure 10:**
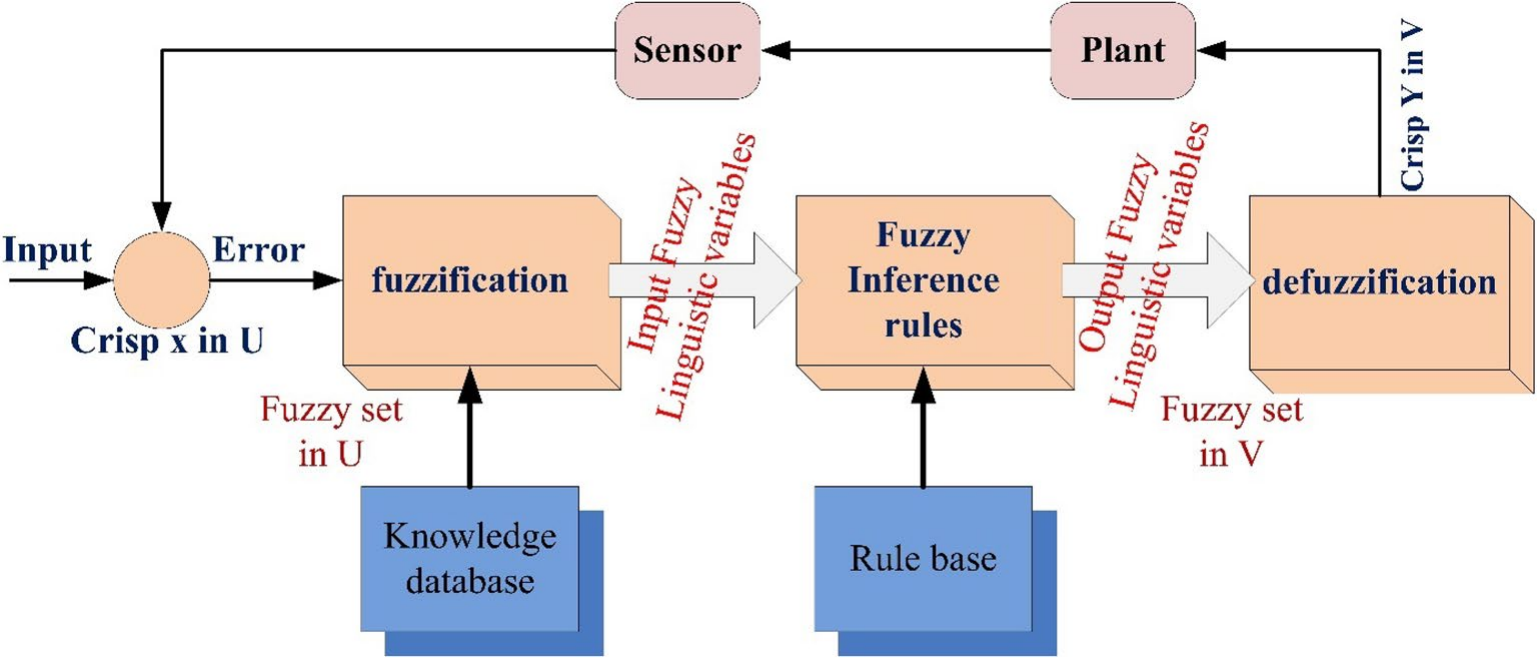
Block diagram of FLC.

Fuzzy logic controller (FLC) was employed to send gate pulses to the VSC and draw harmonics from the signal. FL is a form of thinking that is analogous to human reasoning. This methodology is identical to how humans make decisions. And it encompasses all possible outcomes in between YES and NO. Rules, Fuzzifier, Defuzzifier, and Inference engine are the four main components^91,92^ of FLC as shown in Fig. 10.

Rules- It contains all of the experts rules and IF–THEN conditions^93^ for govern the decision-making system. The field of fuzzy theory has witnessed significant progress, leading to the emergence of several effective techniques for designing and optimizing fuzzy controllers. These advancements often lead to a reduction in the total number of fuzzy rules employed. By utilizing IF–THEN structures, different types of fuzzy rules are formulated to enhance the decision-making process.

15 $${R}_{i}:IF \; e \; is \; {A}_{i}\;AND/OR \; ce\; \mathrm{ is }\;{B}_{i} \;THEN \;{\delta }_{mn} \; is \; {C}_{i}.$$

In Eq. (15) the variables Error (e) and change in error (ce) are normalized within the range [− 1, 1]. Each universe of discourse (Ai, Bi, and Ci) is divided into seven fuzzy subsets: PB (Positive Big), PM (Positive Medium), PS (Positive Small), ZE (Zero), NS (Negative Small), NM (Negative Medium), and NB (Negative Big).

For any combination of e and ce, a maximum of 49 rules are employed. To improve efficiency, the trigonometric membership function was chosen. The Mamdani type is used to formulate rules, as summarized in Table 2.

- *Fuzzifier* This stage transforms the inputs or crisp numbers into fuzzy sets. Sensors can be used to measure the crisp inputs and forward them to the control system for further processing^94^.
- *Inference engine* It quantifies the degree to which fuzzy input and rules are consistent. It will determine which rules to trigger based on the values entered in the input field. The control actions are formed by combining the triggered rules.
- *Defuzzifier* converts fuzzy sets to a single discrete value. There are numerous strategies available, and an expert system may guide you in selecting the most appropriate one^95^.

**Table 2:** Rule base table.

e/ce	NB	NM	NS	Z	PS	PM	PB
**NB**	NB	NB	NB	NB	NM	NS	ZE
**NM**	NB	NB	NB	NM	NS	ZE	PS
**NS**	NB	NB	NM	NS	ZE	PS	PM
**Z**	NB	NM	NS	ZE	PS	PM	PB
**PS**	NM	NS	ZE	PS	PM	PB	PB
**PM**	NS	ZE	PS	PM	PB	PB	PB
**PB**	ZE	PS	PM	PB	PB	PB	PB

**Figure 11:**
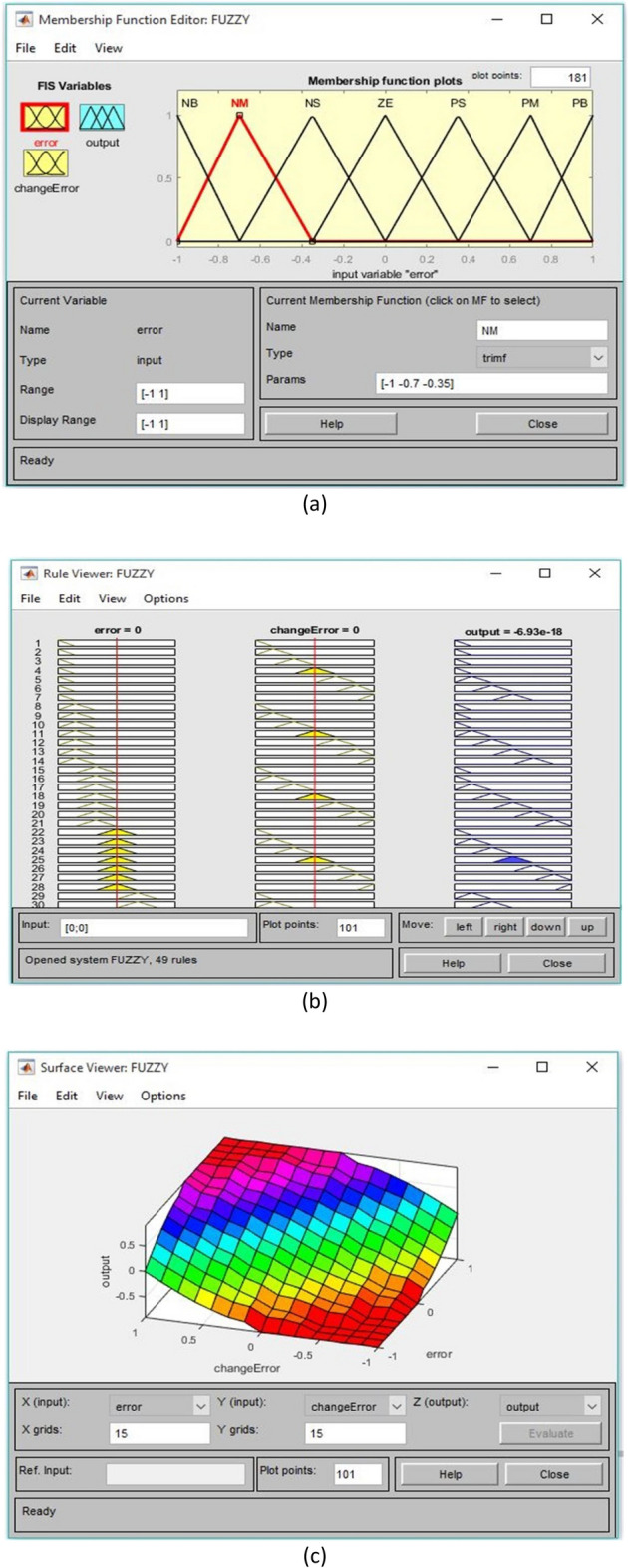
(**a**) Membership functions. (**b**) Fuzzy controller rules. (**c**) Surface plot of the fuzzy layout.

Figure 11a displays the membership function of the fuzzy controller developed in this study. The fuzzy logic controller is fine-tuned using input values ranging from -1 to 1, with dual inputs (e and ce), and a single output value. To create the membership functions, a comprehensive set of input and output values is considered. A total of seven membership functions are examined, resulting in the establishment of 49 rules specifically designed for the fuzzy controller. The designed fuzzy controller rules are visually represented in Fig. 11b, providing an overview of the rule set. Furthermore, Fig. 11c showcases an illustrative surface plot of the fuzzy layout, demonstrating the appropriate configuration of the fuzzy system.

## Results and discussion

The proposed control technique is implemented on a MATLAB/Simulink 9.4.8 (R2018a) platform with 8 GB RAM and an Intel(R) core (TM) i3 processor. The performance of the MF-IUPQC is examined under voltage sag, swell, and harmonic conditions using the PI and FUZZY-controlled MF-IUPQC. The simulation parameters are tabulated in Table 3.

**Table 3:** Simulation parameters.

Parameter	Value
Supply voltage of Feeder1 and Feeder2	11,000 V
Supply frequency	50 Hz
DC link capacitor	2500 µF
DC link voltage	1000 V
Battery capacity	58.5 Ah
BESS nominal voltage	576 V
Membership functions	7
Fuzzy Rules	49
Type of MF	Triangular
Plot points	101
Number of Epoch	100

**Case 1**: **Performance of the system under voltage sag and swell conditions**

**Figure 12:**
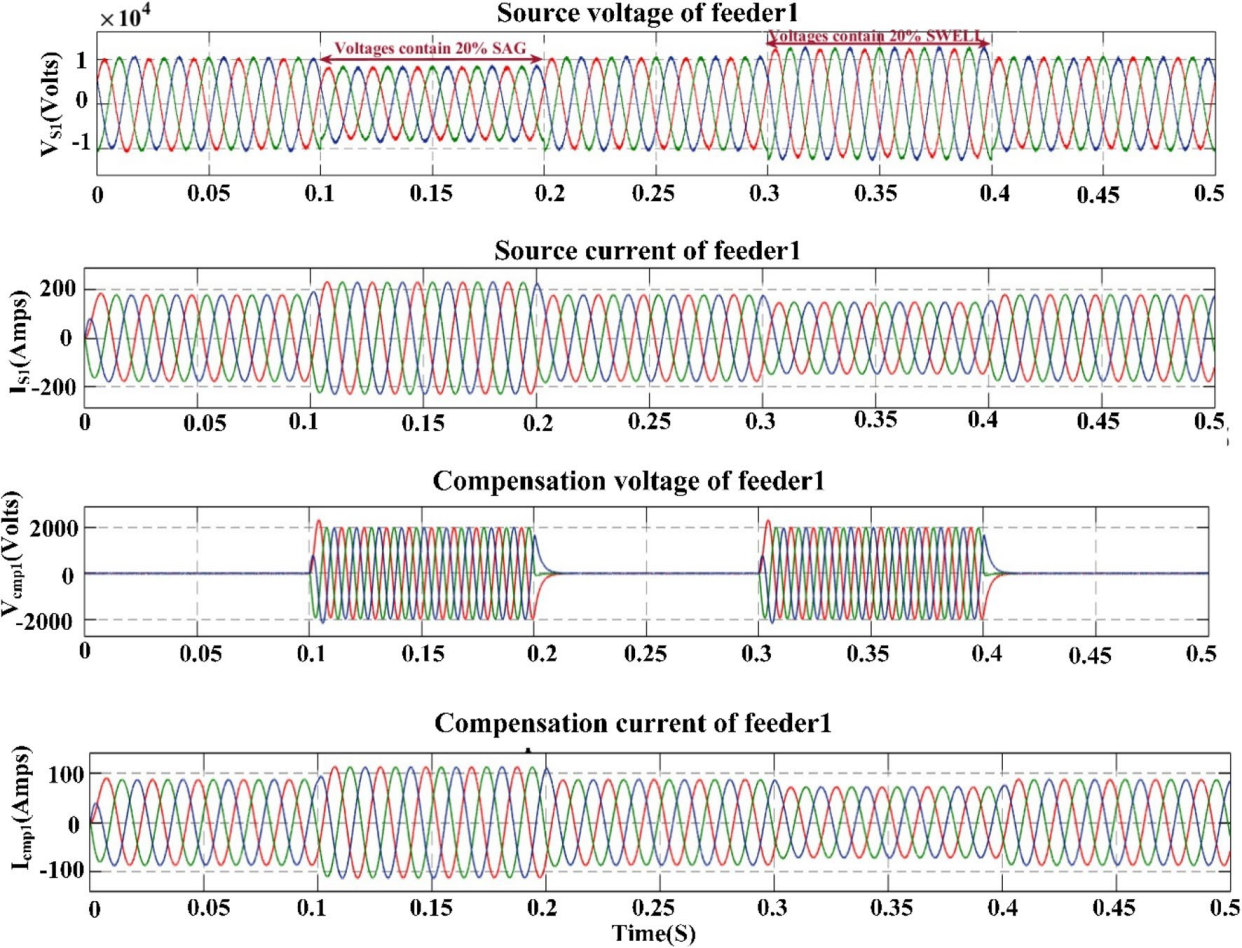
Voltage and current waveforms of first feeder during sag and swell.

**Figure 13:**
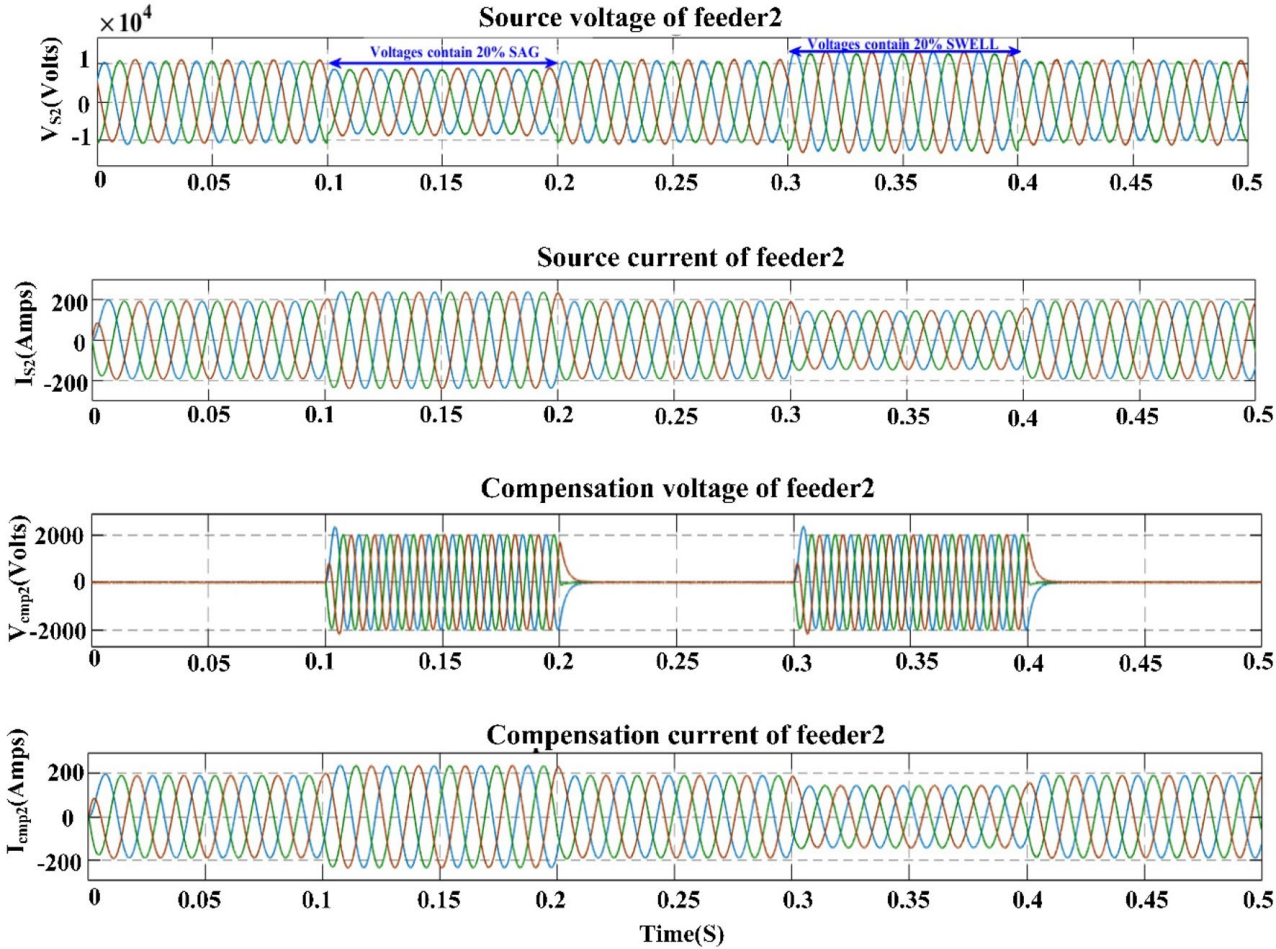
Voltage and current waveforms of second feeder during sag and swell.

**Figure 14:**
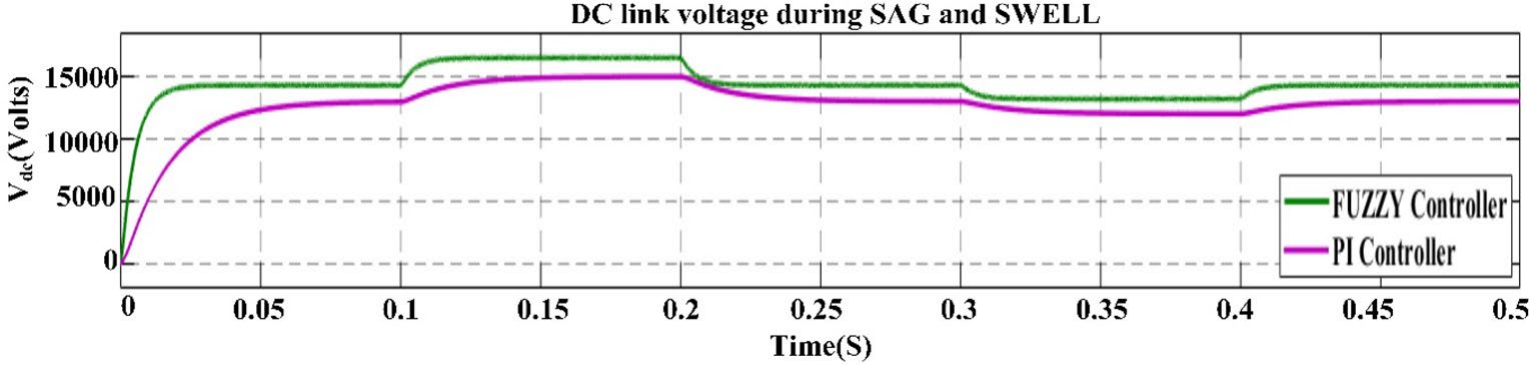
DC-Link voltage during sag and swell.

The MF-IUPQC system experiences voltage sag and swell lasting between 0.1 and 0.2 and 0.3 and 0.4 s, respectively. The source voltages, injected voltages, and currents of the two circuits are depicted in Figs. 12, 13, and 14. During voltage sag, the system voltage decreases by 0.2 per unit (p.u.) and increases by 0.2 p.u. during voltage swell. During voltage sag and voltage swell, the magnitude of the current increases and decreases simultaneously. To counteract these effects, series voltage source converters (VSCs) inject compensating voltages, while shunt VSCs inject compensating currents. The system responds to periods of low voltage by injecting reactive power from the DC-link capacitor. The voltage waveform of the DC-link capacitor showcases the remarkable dynamic response of the system. Table 4 provides a comparison of various variables, including rise time, slew rate, settling time, RMS voltage, peak value, and peak time. By injecting compensating voltages and currents, the MF-IUPQC system effectively mitigates voltage sags and surges, exhibiting outstanding performance in terms of response variables such as rise time, slew rate, settling time, RMS voltage, peak value, and peak time.

**Table 4:** DC Link voltage comparison of MF-IUPQC with PI and Fuzzy controller.

Parameters	With PI controller	With fuzzy logic controller
Rise time (ms)	36.033	10.826
Slew rate (/μs)	0.291	1.049
Overshoot	15.525%	0.192%
RMS	12.89 kV	14.41 kV
Peak value	15 kV	16.4 kV
Peak-time (sec)	0.199	0.185

**Case 2**: **Performance of the system under voltage harmonics**

**Figure 15:**
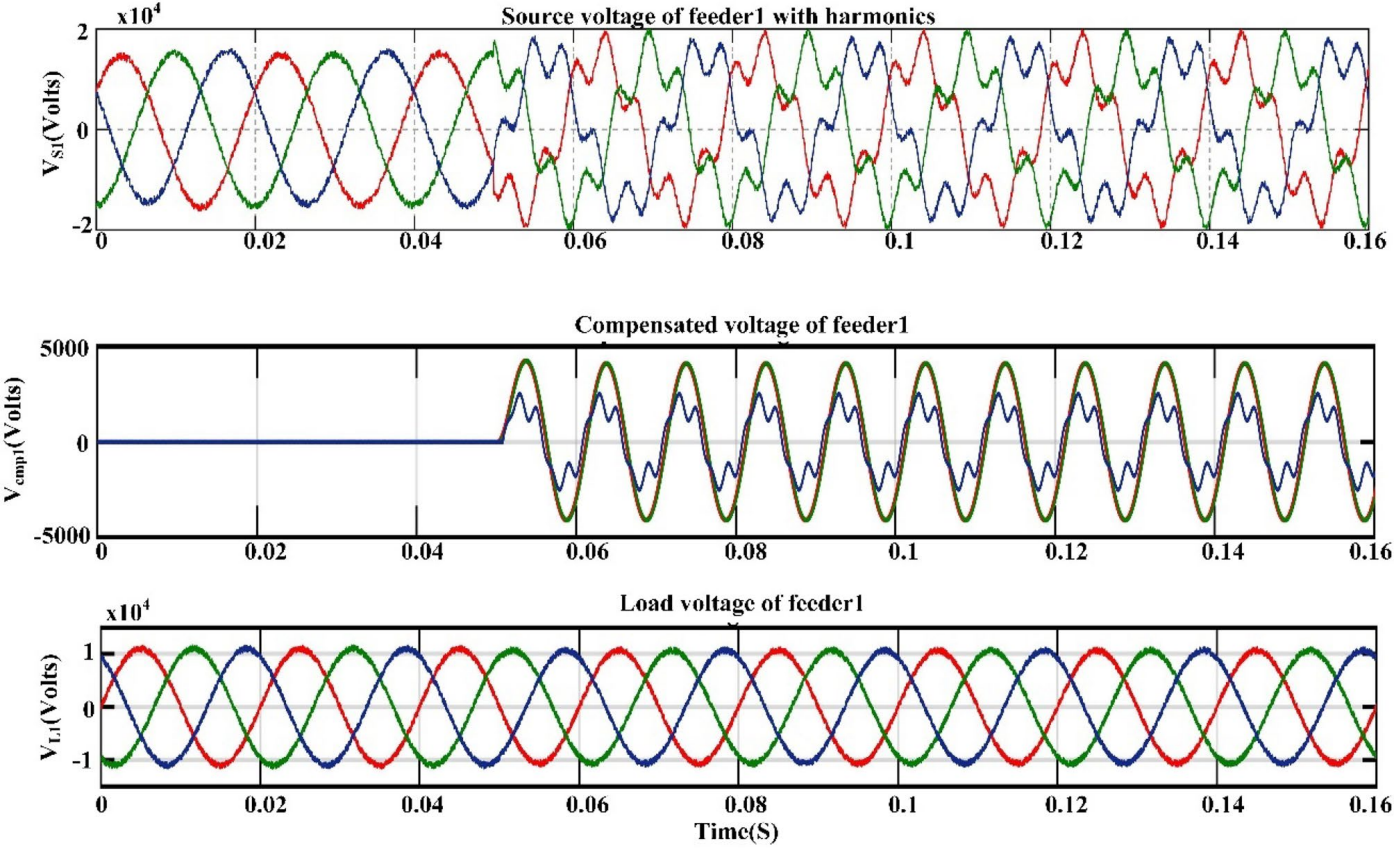
Waveforms of feeder1 during voltage harmonics.

**Figure 16:**
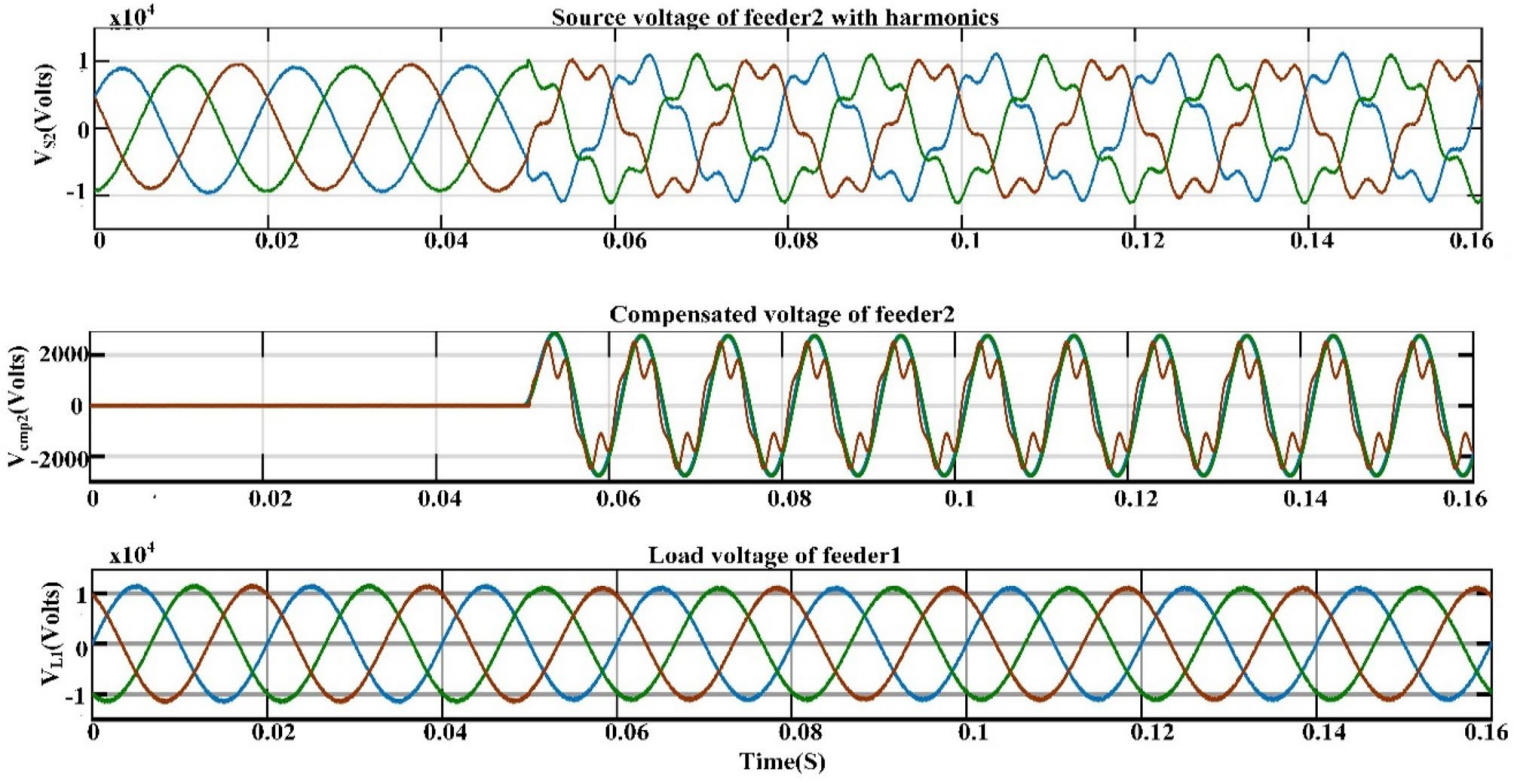
Waveforms of feeder2 during voltage harmonics.

**Figure 17:**
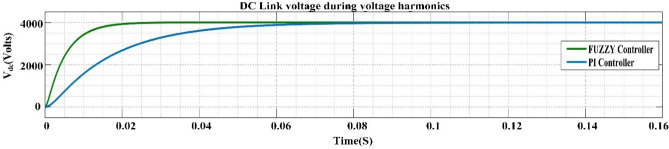
DC-Link voltage during voltage harmonics.

**Figure 18:**
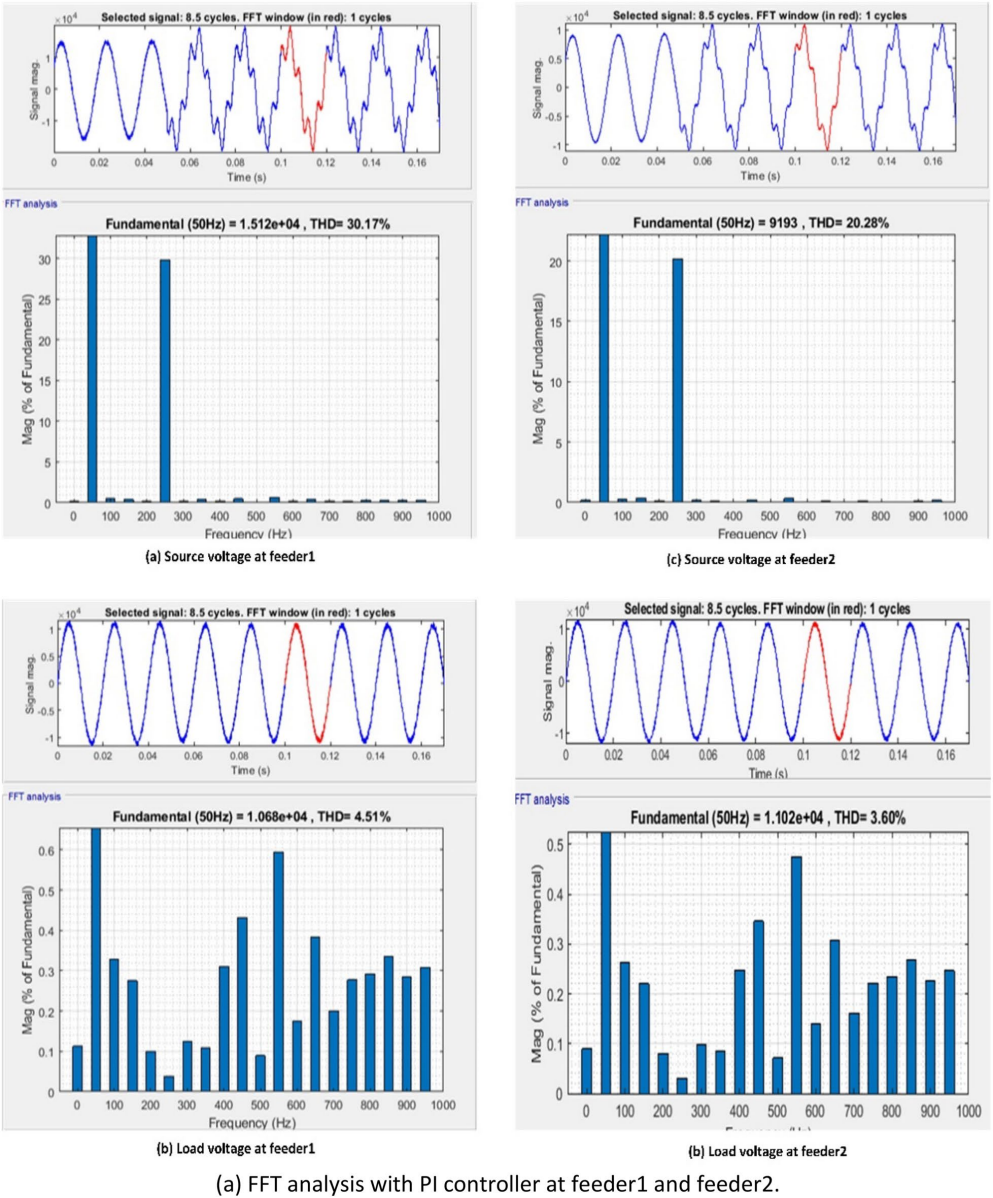

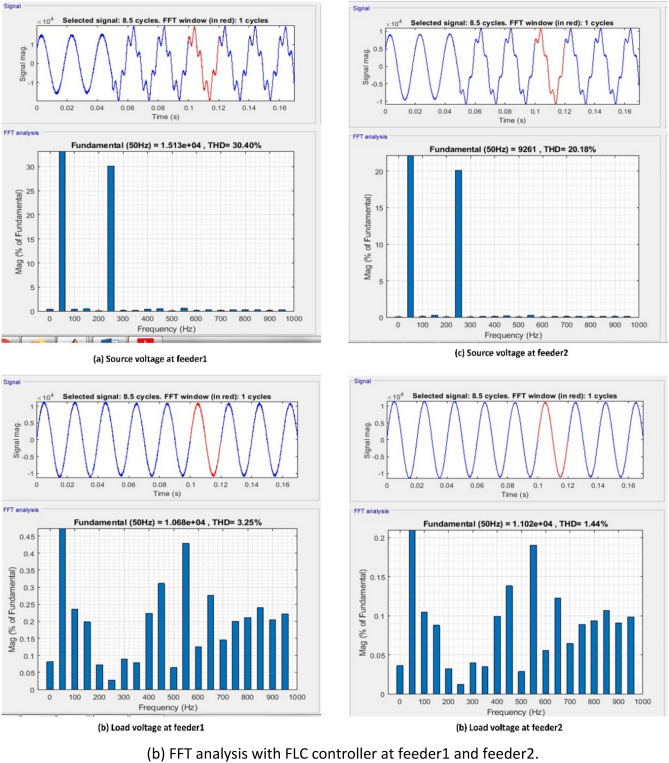
FFT analysis during voltage harmonics.

To simulate voltage irregularities, intentional generation of harmonics of the third and fifth orders is implemented in both feeders. The first feeder introduces 15% third-order harmonics and 20% fifth-order harmonics starting at t = 0.05 s, while the second feeder, which supplies the loads, has 15% third-order harmonics and 15% fifth-order harmonics. The waveforms of source, compensated, and load voltages for both feeders with fuzzy controllers are depicted in Figs. 15, 16, and 17. In these waveforms, the source voltage waveforms of both feeders exhibit harmonics, while the load voltage waveforms remain sinusoidal due to the compensation voltage provided by the series voltage source converters (VSCs) of the Integrated Unified Power Quality Conditioner (IUPQC). Figure 18a,b display the results of an FFT analysis with the THD values of the PI and the fuzzy controller, respectively. The compensation significantly reduces the total harmonic distortion (THD) from the source to the load, as demonstrated in Table 5. Feeder 1 experiences a decrease in THD from 30.4 to 3.25%, and Feeder 2 sees a decrease from 20.18 to 1.44%. Furthermore, the DC link voltage waveform indicates that the fuzzy controller effectively handles voltage harmonics within the system. This confirms the controller's capability to mitigate and manage harmonics, enhancing the overall performance and stability of the system.

**Table 5:** THD during voltage harmonics.

Controller	THD during voltage harmonics			
	Over Feeder1		Over Feeder2	
	Source side	Load side	Source side	Load side
PI	30.17	4.51	20.28	3.60
Fuzzy	30.4	3.25	20.18	1.44

**Case 3**:** Performance of the system under current harmonics**

**Figure 19:**
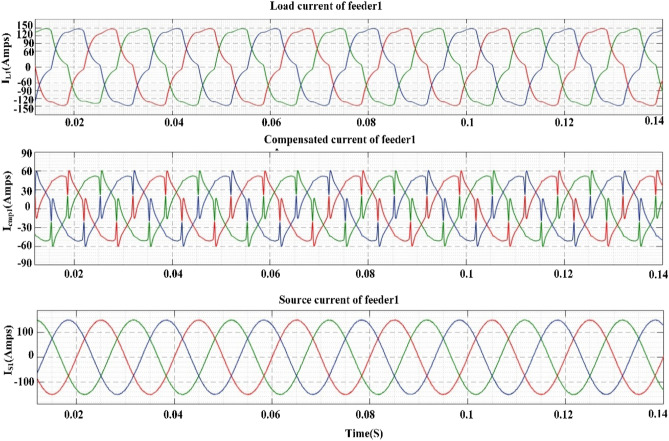
Waveforms of feeder1 during current harmonics.

**Figure 20:**
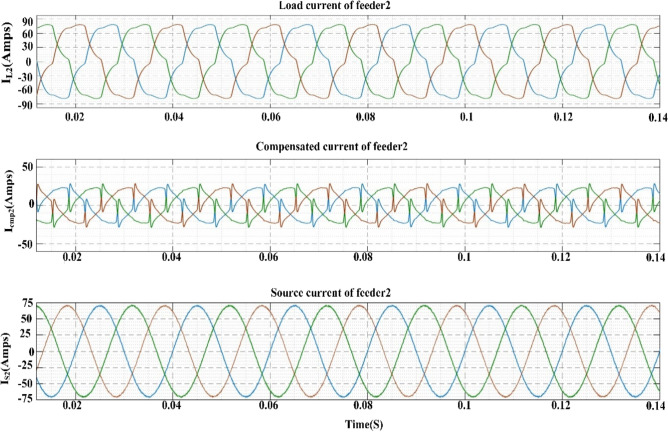
Waveforms of feeder2 during current harmonics.

**Figure 21:**
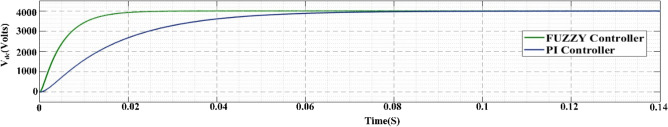
DC-Link voltage during current harmonics.

**Figure 22:**
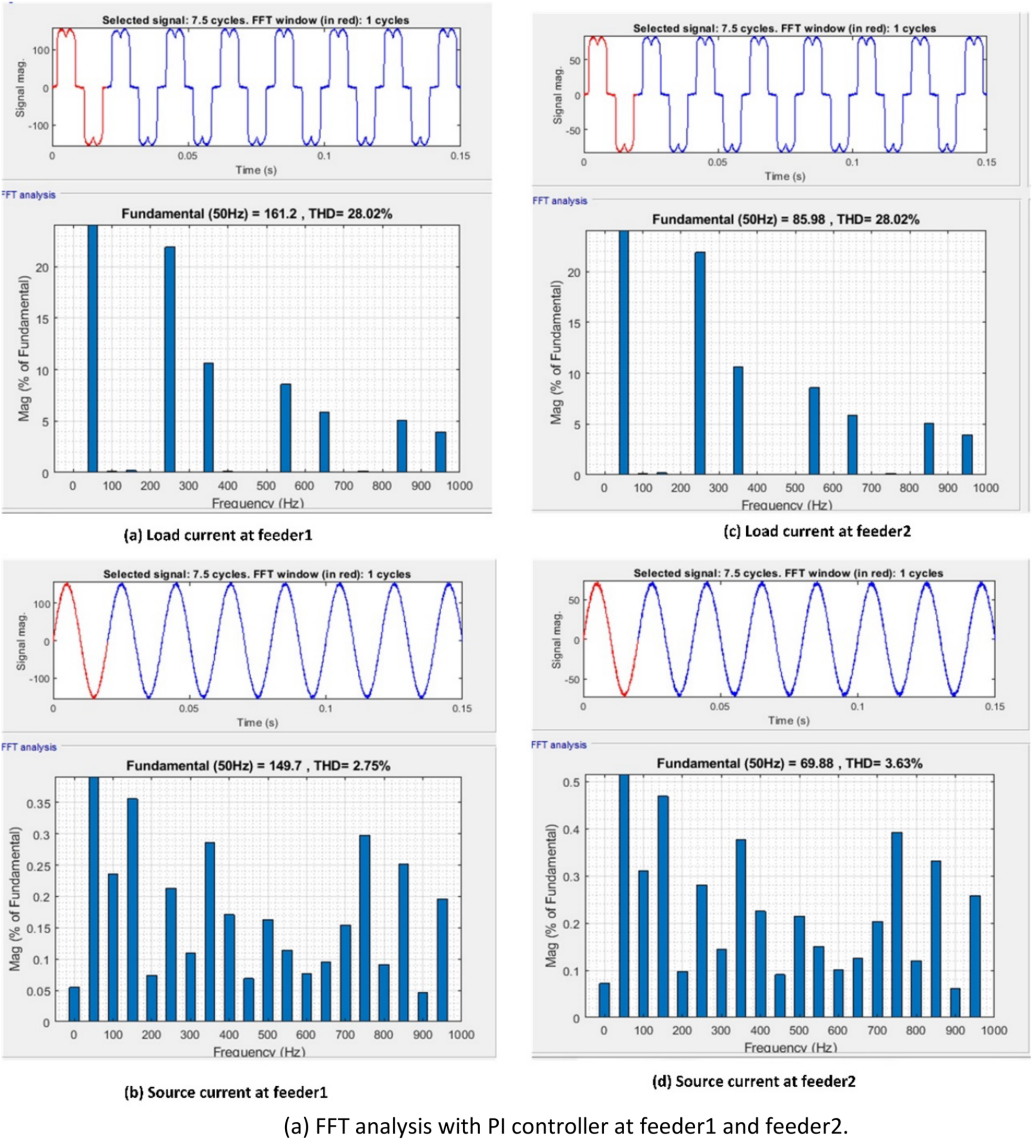

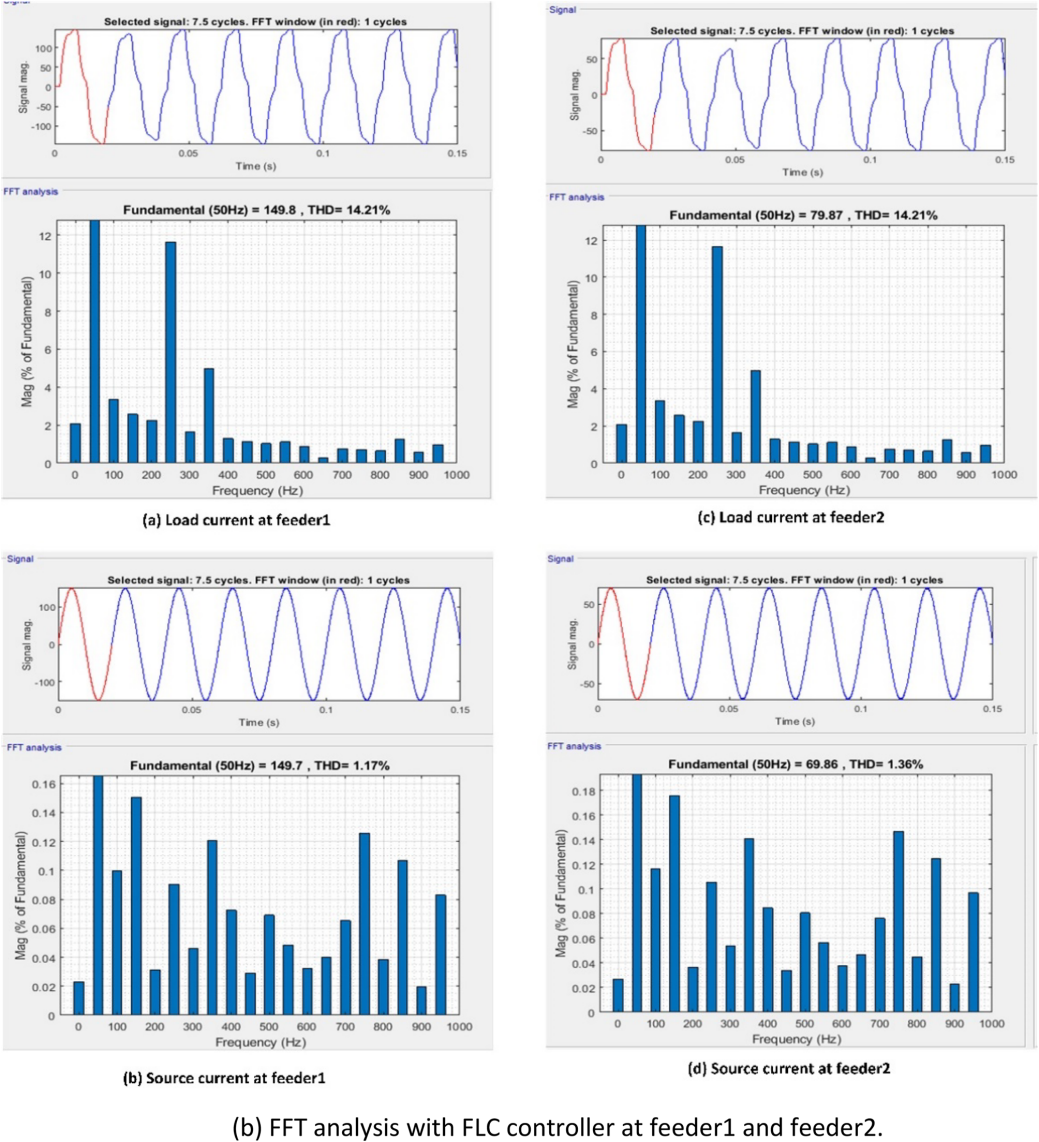
FFT analysis during current harmonics.

A nonlinear load is produced by the presence of a diode bridge rectifier, resulting in distorted current waveforms. The diode bridge rectifier on feeders 1 and 2, respectively, is responsible for supplying the resistive loads of 100 ohms and 200 ohms that are present in this system. The waveforms of the load current, the compensated current, and the source current for both feeders equipped with fuzzy control systems are depicted in Figs. 19, 20, and 21, respectively. The load currents of both feeders are distorted due to nonfundamental harmonics. To mitigate current harmonics, the Integrated Unified Power Quality Conditioner (IUPQC) shunt voltage source converters (VSCs) inject compensating currents into the system. This results in nearly sinusoidal source current waveforms for both feeders, effectively reducing the load-to-source THD.Fig. 22(a) and Fig. 22(b) display the results of an FFT analysis with the THD values of the PI and the fuzzy controller, respectively. Table 6 demonstrates the reduction in THD from the load current to the source current using THD values. The THD is reduced from 14.21 to 1.17% for the first feeder and from 14.21 to 1.36% for the second feeder. This indicates that the fuzzy controller is effective at reducing the system's current harmonics. In addition, the performance of the fuzzy controller is reflected in the stable DC link voltage despite the presence of current harmonics in the system. It can be seen from the DC link voltage that the fuzzy controller works effectively with the current harmonics in the system.

**Table 6:** THD during current harmonics.

Controller	THD of current from load to source			
	Over Feeder1		Over Feeder2	
	Load side	Source side	Load side	Source side
PI	28.02	2.75	28.02	3.63
Fuzzy	14.21	1.17	14.21	1.36

### Limitations of FLC based IUPQC

Although the fuzzy logic based Interline Unified Power Quality Conditioner (IUPQC) is an effective method to mitigate power quality disturbances, it is important to understand the constraints within which it operates. Firstly, its complexity can be a challenge, particularly when implementing it in large-scale systems. Competence is required in the design of the fuzzy rules and membership functions, and extensive tuning may be required. Knowledge is required in the design of the fuzzy rules and membership functions, and extensive tuning may be required. Scalability can also be a problem since the performance of FLC-based controllers may not change smoothly in power systems that are larger or more complex. In addition, the dependence of FLC on predetermined linguistic rules and membership functions makes it dependent on the specific model being used. If the model employed by the system fails to accurately depict the conditions existing in the real world, the performance of the fuzzy logic controller (FLC) may be adversely affected. Furthermore, the system exhibits a restricted capacity for responding to dynamic changes and may encounter difficulties in handling unexpected disruptions. Furthermore, the issue of computational expense arises due to the requirement of real-time calculation in fuzzy logic control (FLC), which has the potential to introduce additional complexity to the system. Although FLC has proven to be an efficient method to handle a range of power quality issues, it is important to recognize that there may be trade-offs when trying to optimize numerous components simultaneously. Because it requires knowledge of fuzzy logic control to implement, it is both time-consuming and difficult to understand. Finally, its performance may be affected by the limited amount of available historical data. In spite of these drawbacks, fuzzy logic based IUPQC can be a useful tool if its limits are properly assessed in accordance with the requirements of a certain application or system.

## Conclusion

This study investigated various challenges associated with the integration of solar and wind energy into the electrical grid and proposed a fuzzy logic-based multi-feeder interline unified power-quality conditioner to mitigate power quality disturbances. The study emphasized the causes, effects, and mitigation technologies associated with the topology of wind-solar systems that are grid-integrated. Through a literature review, various studies were examined, and their findings were incorporated into the manuscript. Literature analysis found that DVRs quickly correct voltage sags and swells, ensuring a stable supply to sensitive equipment. Stability is improved through STATCOM voltage regulation and reactive power. By increasing current quality and minimizing reactive power, APFs reduce harmonic distortion. Energy storage systems (ESS) perform a vital role in enhancing reliability and harmonic control. With series and shunt correction, UPQCs manage sags, swells, harmonics, and imbalances. Finally, the MF-IUPQC's implementation of a fuzzy logic controller (FLC) was discussed. This controller addresses PQ disturbances such as voltage and current fluctuations and harmonic distortions effectively. Utilizing the FLC in the MF-IUPQC system resulted in enhanced load voltage regulation, stable DC-link voltage, and efficient harmonic compensation. Total harmonic distortion (THD) values for both inputs of the proposed system were lower than those achieved by other controllers. Source-to-load total harmonic distortion (THD) dropped from 30.4 to 3.25% in Feeder 1 and from 20.18 to 1.44% in Feeder 2. Furthermore, the fuzzy controller reduced THD from load current to source current from 14.21 to 1.17% for the first feeder and from 14.21 to 1.36% for the second feeder. Future research can investigate enhanced Model Predictive Controller (MPC) models to better integrate solar and wind energy into the grid and maintain a sustainable and reliable electricity supply. These MPC models can provide a new viewpoint for evaluating accuracy and robustness, allowing the proposed FLC-based solutions to be improved. There is hope that technological progress like this can improve the effectiveness and dependability of renewable energy systems that are connected to the grid.

## Data Availability

All data generated or analyzed during this study are included in this published article. Further if someone wants to request the data from this study should contact corresponding author or first author.

## Abbreviations

ANFIS: Adaptive neuro fuzzy inference system
APF: Active power filter
ANF: Adaptive notch filters
BESS: Battery energy storage system
CHB: Cascaded H-bridge
DFIG: Double fed induction generator
DPC: Direct power controller
DVR: Dynamic voltage restorer
DSTATCOM: Distribution static compensator
ESS: Energy storage systems
FACTS: Flexible alternating current transmission system
FCL: Fault current limiters
FAFLC: Firefly asymmetrical FLC
FLLs: Frequency-locked loops
GHG: Greenhouse gases
GIPVS: Grid integrated photovoltaic systems
GISES: Grid integrated solar energy systems
GIWES: Grid integrated wind energy system
GSC: Grid-side converter
GTI: Grid-tie inverter
HCS: Hill climb searching
HRES: Hybrid renewable energy system
IG: Induction generator
Inc: Cond- Incremental conductance algorithm
IPC: Indirect power controller
LG: Line to ground
LLG: Line to line to ground
LS-PO: Large-step P&O
LVRT: Low voltage ride through
MLI: Multilevel inverter
MPPT: Maximum power point tracking
MSC: Machine-side converter
NPC: Neutral point clamped
P&O: Perturb and observe
PCC: Point of common coupling
PF: Power factor
PMSG: Permanent magnet synchronous generator
PQ: Power quality
PRI: Proportional-resonant-integral
PV: Photovoltaic
PWM: Pulse width modulation
RES: Renewable energy sources
RESs: Renewable energy systems
SCIG: Squirrel cage induction generator
SC: Super capacitor
SOGI: Second-order generalised integrators
S-PO: Small step P&O
STATCOM: Static compensator
THD: Total harmonics distortion
UPQC: Unified power quality controller
VSI: Voltage source inverter
VS-PO: Variable-step Perturb and Observe
WECS: Wind energy conversion systems
WT: Wind turbine
